# Discovery of novel 2-(4-(benzyloxy)-5-(hydroxyl) phenyl) benzothiazole derivatives as multifunctional MAO-B inhibitors for the treatment of Parkinson’s disease

**DOI:** 10.1080/14756366.2022.2159957

**Published:** 2023-02-02

**Authors:** Zhongcheng Cao, Xingyue Wang, Tianlong Zhang, Xianwu Fu, Fan Zhang, Jiang Zhu

**Affiliations:** a School of Pharmacy, North Sichuan Medical College, Nanchong, China; b Sichuan Key Laboratory of Medical Imaging, School of Pharmacy and Nanchong Key Laboratory of MRI Contrast Agent, North Sichuan Medical College, Nanchong, China

**Keywords:** Parkinson’s disease, 2-(4-(benzyloxy)-5-(hydroxyl) phenyl) benzothiazole derivatives, MAO-B inhibitors, anti-neuroinflammatory agents, multifunctional anti-PD agents

## Abstract

To discover novel multifunctional agents for the treatment of Parkinson’s disease, a series of 2-(4-(benzyloxy)-5-(hydroxyl) phenyl) benzothiazole derivatives was designed, synthesized and evaluated. The results revealed that representative compound **3h** possessed potent and selective MAO-B inhibitory activity (IC_50_ = 0.062 µM), and its inhibitory mode was competitive and reversible. Additionally, **3h** also displayed excellent anti-oxidative effect (ORAC = 2.27 Trolox equivalent), significant metal chelating ability and appropriate BBB permeability. Moreover, **3h** exhibited good neuroprotective effect and anti-neuroinflammtory ability. These results indicated that compound **3h** was a promising candidate for further development against PD.

## GRAPHICAL ABSTRACT

**Figure:**
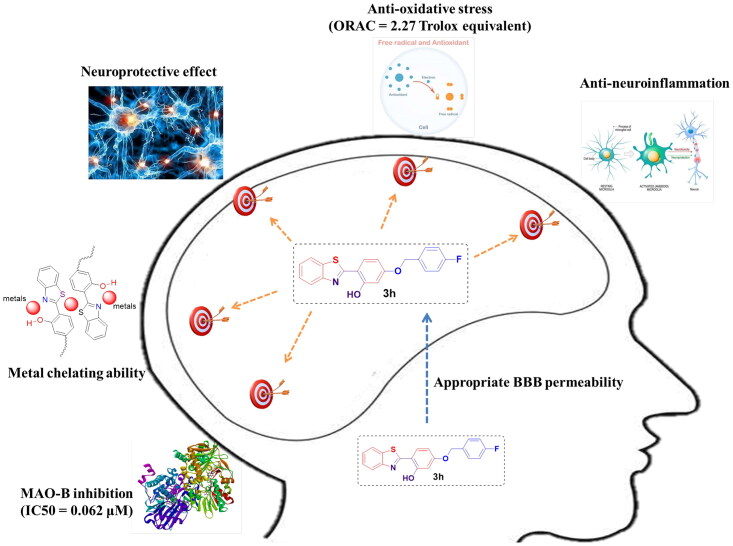

## Introduction

Parkinson’s disease (PD), the second common neurodegenerative disease, is characterized by the loss of dopamine neurons and formation of Lewy bodies (LB)^1^. The symptoms of PD include motor symptoms (tremor, rigidity, bradykinesia, and postural instability) and non-motor symptoms (anosmia, constipation, sleep disorders, dementia, and depression), causing immense agony to the patients and caregivers^2^. Nowadays, more than 6.1 million people are suffering from PD, and this figure is increasing rapidly which makes PD become the fastest-growing neurological disorder in prevalence, disability, and deaths^3^. However, there is no drug or therapy that can arrest or reverse the progression of PD^4^. Therefore, it is urgent to discovery novel anti-PD agents.

The pathogenesis of PD is still not clear, but studies have revealed that several factors played important role in this process, such as abnormal aggregation of *α*-synuclein, increase in oxidative stress, neuroinflammation, bio-metals dyshomeostasis, decline in dopamine (DA) level and so on^5^. The dopamine neurons in the brain of PD patients are pathologically degenerated, while the level of monoamine oxidase B (MAO-B) is conversely increased^6^. Because DA was predominantly produced by dopamine neurons and catalytically decomposed by MAO-B, these pathological changes could significantly reduce the DA level in PD patients and lead corresponding motor and non-motor syptoms^7^. Besides, the deamination of DA catalysed by MAO-B could produce a large amount of reactive oxygen species (ROS) and reactive nitrogen species (RNS), which would induce the increase in oxidative stress^8^. On the one hand, the overproduced ROS and RNS can damage the lipid, protein and DNA, leading the death of cells; on the other hand, these reactive species can also promote the aggregation of *α*-synuclein and induce neuroinflammation, which have the ability to aggravate the oxidative stress *vice versa*^9^. Additionally, bio-metals dyshomeostasis also contributes to the increase in oxidative stress level^10^. In detail, iron ions are abnormally elevated in the striatum of PD patients, and these iron ions can accelerate the production of ROS through Fenton reaction^11^. Besides, iron ions can also induce the death of cells through ferroptosis.^12^ Copper ion also has the ability to induce Fenton reaction, while its concentration is comparatively low in the brain of PD patients^13^. Therefore, drugs which possess MAO-B inhibitory activities, antioxidant abilities and metal chelating effects are necessary in PD patients.

Neuroinflammation is another hallmarker of PD^14^. Under normal conditions, neuroinflammation helps us to defense against harmful invasion and eliminate waste, but, when neuroinflammation is chronic, it triggers neuronal damage^15^. Unfortunately, chronic neuroinflammation is clearly existed in the brain of PD patients^16^. The microglias in the brain of PD patients are pathologically activated, and then excess inflammatory cytokines would be released, like tumour necrosis factor-α (TNF-α), interleukin-1*β* (IL-1*β*), nitric oxide (NO), which have the ability to induce the dopaminergic neuronal death, increase in oxidative stress and *α*-synuclein aggregation^17^. So, inhibiting neuroinflammation is an efficient way to cure PD.

PD is a complex disease, so drugs which can only intervene one single target are not efficient to cure PD^18^. Current studies revealed that using “multi-target-directed ligands” (MTDLs) strategy to design small molecules which could interact with multiple targets of complex disease possessed natural advantages^19^. Especially, the agents with MAO-B inhibitory activity and other activities may have the ability to reverse the progression of PD while relieving the symptoms^20^.

**Figure 1.**
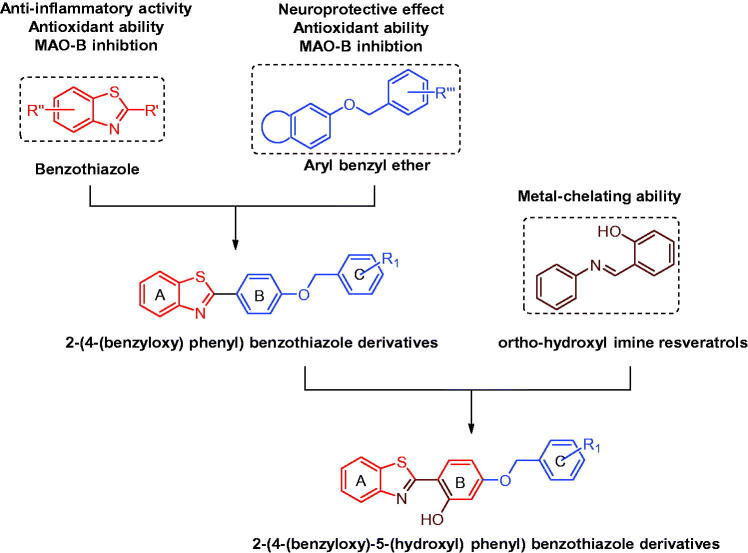
Design strategies of 2-(4-(benzyloxy)-5-(hydroxyl) phenyl) benzothiazole derivatives.

Benzothiazole is a versatile bicyclic heterocycle and benzothiazole derivatives exhibited various biologic activities, such as anti-inflammatory activity, antimicrobial activity, anticonvulsant activity, antioxidant activity and so on^21–24^. Recent studies also revealed that some benzothiazole derivatives possessed good anti-PD effects. In particular, the indole-substituted benzothiazoles designed by Nam et al. generally exhibited excellent MAO-B inhibitory activities, and the representative compound even displayed a good therapeutic effect on Parkinsonian motor symptom in MPTP-induced PD model^25^. Therefore, benzothiazole is an ideal scaffold for the discovery of novel anti-PD agents. Furthermore, studies revealed that aryl benzyl ether is a multifunctional pharmacophore, and aryl benzyl ether derivatives showed many anti-PD activities, such as MAO-B inhibition, antioxidant activity, and neuroprotective effect^26–29^. Therefore, benzothiazole skeleton was hybrided with aryl benzyl ether pharmacophore to afford a series of 2-(4-(benzyloxy) phenyl) benzothiazole derivatives firstly. Interestingly, the obtained derivatives possess much similar scaffold with imine resveratrols, while some imine resveratrol derivatives also displayed good metal-chelating ability when the *ortho*-position on the benzene ring was substituted by hydroxyl group, since this structure was similar with clioquinol (a metal chelator)^30^. Therefore, hydroxyl group was further introduced to the *ortho*-position on the B benzene ring of 2-(4-(benzyloxy) phenyl) benzothiazole derivatives. Taken together, a series of 2-(4-(benzyloxy)-5-(hydroxyl) phenyl) benzothiazole derivatives were designed, and these derivatives were expected to act as MAO-B inhibitors with antioxidant activities, metal chelating abilities, neuroprotective effects, anti-neuroinflammatory activities and BBB permeation abilities. The design strategy is depicted in Figure 1.

## Results and discussion

### Chemistry

**Scheme 1.**
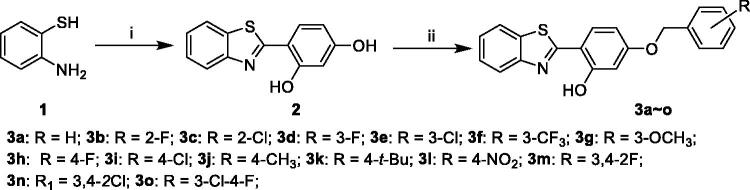
Synthesis of 2-(4-(benzyloxy)-5-(hydroxyl) phenyl) benzothiazole derivatives (**3a–o**). *Reagents and conditions*: (i) 2,4-dihydroxybenzaldehyde, Na_2_S_2_O_5_, DMF; (ii) corresponding substituted benzyl chlorides or benzyl bromides, NaHCO_3_, KI, CH_3_CN, at 60 °C for 30 h.

2-(4-(benzyloxy)-5-(hydroxyl) phenyl) benzothiazole derivatives were synthesised by using 2-aminothiophenol (**1**) as starting material (Scheme 1). The condensation of compound **1** with 2,4-dihydroxybenzaldehyde in the presence of Na_2_S_2_O_5_ afforded 2-(2,4-(dihydroxyl) phenyl) benzothiazole (**2**)^31^. Then, the subsequent reaction of key intermediate (**2**) with corresponding benzyl chlorides or benzyl bromides under the condition of NaHCO_3_/KI in CH_3_CN produced 2-(4-(benzyloxy)-5-(hydroxyl) phenyl) benzothiazole derivatives^32^. All the target compounds were characterised by the ^1^H NMR, ^13^C NMR, and ESI-MS in this study. In addition, the purity of target compounds was determined by high-performance liquid chromatography (HPLC) analysis to be over 97%.

### Pharmacology

#### Evaluation of MAOs inhibitory activities

The MAOs inhibitory activities of 2-(4-(benzyloxy)-5-(hydroxyl) phenyl) benzothiazole derivatives were measured by kynuramine method^33^, with safinamide, rasagiline, iproniazid, and clorgyline as positive compounds. The result was shown in Table 1. Noticeably, all the derivatives exhibited excellent MAO-B inhibitory activities, while their MAO-A inhibitory effects were comparatively weak, so these derivatives are selective MAO-B inhibitors. Among the target derivatives, representative compound **3h** displayed the most potent MAO-B inhibitory activity with an IC_50_ value of 0.062 µM, which is stronger than the IC_50_ value of rasagiline (IC_50_ = 0.0953 µM) but slightly weaker than the IC_50_ value of safinamide (IC_50_ = 0.0572 µM). Besides, compound **3n** showed the greatest MAO-A inhibition with an IC_50_ value of 9.80 µM.

**Table 1.** Inhibitory effects on MAOs and antioxidative activities of 2-(4-(benzyloxy)-5-(hydroxyl) phenyl) benzothiazole derivatives and reference compounds *in vitro.*

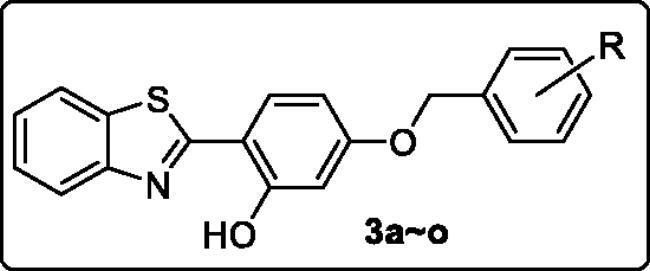				
Compounds	R	%Inhibition or IC_50_ (μM)^a^		ORAC^b^
		MAO-A	MAO-B	
**3a**	H	24.83 ± 0.11%	3.933 ± 0.259	2.13 ± 0.05
**3b**	2-F	14.25 ± 0.07%	1.674 ± 0.146	2.32 ± 0.02
**3c**	2-Cl	19.58 ± 0.06%	2.895 ± 0.031	2.26 ± 0.09
**3d**	3-F	18.41 ± 0.05%	0.526 ± 0.024	2.24 ± 0.10
**3e**	3-Cl	31.60 ± 0.28%	1.049 ± 0.083	2.31 ± 0.07
**3f**	3-CF_3_	n.a.	0.951 ± 0.062	2.25 ± 0.07
**3g**	3-OCH_3_	n.a.	1.523 ± 0.097	2.30 ± 0.04
**3h**	4-F	39.73 ± 0.54%	0.062 ± 0.005	2.27 ± 0.08
**3i**	4-Cl	47.14 ± 0.21%	0.314 ± 0.010	2.23 ± 0.06
**3j**	4-CH_3_	34.32 ± 0.09%	1.942 ± 0.078	2.39 ± 0.14
**3k**	4-*t*Bu	n.a.	3.407 ± 0.032	2.48 ± 0.13
**3l**	4-NO_2_	25.16 ± 0.13%	0.784 ± 0.028	2.14 ± 0.06
**3m**	3,4-2F	47.52 ± 0.36%	0.423 ± 0.009	2.24 ± 0.11
**3n**	3,4-2Cl	9.80 ± 0.19	0.970 ± 0.011	2.21 ± 0.09
**3o**	3-Cl-4-F	49.91 ± 0.53%	0.521 ± 0.007	2.27 ± 0.05
Safinamide	–	24.9 ± 0.04%	0.0572 ± 0.002	1.12 ± 0.01
Rasagiline	–	1.29 ± 0.06	0.0953 ± 0.001	NT
Iproniazid	–	1.87 ± 0.02	1.23 ± 0.037	NT
Clorgyline	–	0.0025 ± 0.0003	3.66 ± 0.09	NT

n.a.: no active, meaning the percent inhibition is <5.0%; NT: not tested.

^a^Percent inhibition was determined at the concentration of 10.0 μM and the IC_50_ value is the concentration when the compound inhibit enzyme activity by 50%.

^b^Data are expressed as Trolox equivalents.

The structure-activity relationship (SAR) analysis revealed that the substituent groups and their number as well as position played important role in the MAO-A/B inhibitory activities. Obviously, compared with compound **3a**, derivatives **3b–3o** showed stronger MAO-B inhibition, indicating the introduction of substituent groups on the benzyloxy ring is favourable for MAO-B inhibition. Besides, derivatives with fluoro substitution showed better MAO-B inhibition than compounds which bear other substituent groups on the same position, while derivatives with chlorine substitution exhibited more potent MAO-A inhibitory activities. This result reflected that the fluoro substitution on the benzyloxy ring was good for the interaction of target compounds with MAO-B, and chlorine substitution was beneficial for the derivatives to occupy the catalytic centre of MAO-A. Additionally, it is noticeable that derivatives (**3h** and **3i**) with fluoro substitution or chlorine substitution on the *para*-position of benzyloxy ring exhibited stronger MAO-A/B inhibitory activity than compounds with fluoro substitution or chlorine substitution on the *ortho*-position and *meta*-position. This phenomenon revealed that it is more favourable for MAO-A/B inhibition when the halogenation occurs on the *para*-position of benzyloxy ring. Furthermore, **3m** and **3n** displayed superior MAO-A inhibitory activities than **3d**, **3e**, **3h**, and **3i**, so the dihalogenation on the 3,4-position of benzyloxy ring is favourable for the improvement of MAO-A inhibitory activities. Interestingly, the MAO-A/B inhibitory activities and SAR analysis results of these compounds are much similar with those of coumarin derivatives reported by Secci et al.^34^, which may provide clue to find new MAOs inhibitors.

#### Kinetic study

**Figure 2.**
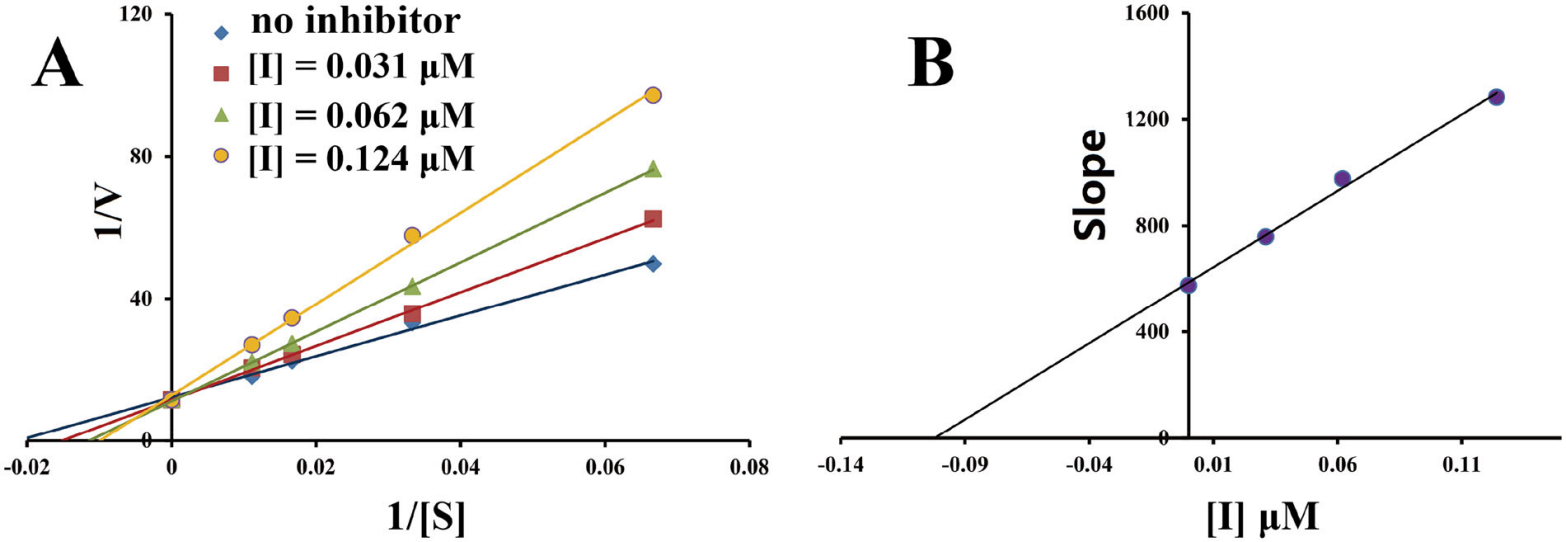
Kinetic study of MAO-B inhibitory mechanism by compound **3h**. (A) Lineweaver-Burk plots of MAO-B activities in the absence and presence of various concentrations of **3h** (0.031, 0.062, and 0.124 μM), (B) the slopes of the Lineweaver-Burk plots *vs.* the **3h** concentrations.

Kinetic study^35^^,^^36^ was conducted to figure out the MAO-B inhibitory mechanism of 2-(4-(benzyloxy)-5-(hydroxyl) phenyl) benzothiazole derivatives, with **3h** as the test compound. The catalytic rates of MAO-B were measured at four concentrations of kynuramine (15, 30, 60, and 90 µM), and the Lineweaver-Burk plots were thus constructed in the absence or presence of three concentrations of **3h** (2 × IC_50_, IC_50_, and 1/2 IC_50_). The graphical of the reciprocal Lineweaver-Burk plots was depicted in Figure 2(A). It is noticeable that both the slopes (decreased *V_max_*) and intercepts (higher *K_m_*) were increased when the concentration of **3h** was elevated, and these straight lines intersected at Y axis. This result revealed that the inhibition pattern of **3h** was competitive, which provides the support for that **3h** is a reversible MAO inhibitor. Besides, the graph obtained from the slopes of the Lineweaver-Burk plots *vs.* inhibitor concentration (Figure 2(B)) revealed the competitive inhibition constant (*Ki*) of **3h** was 0.102 µM, as the x-axis intercept is −*Ki*.

#### Reversibility study

To figure out the MAO-B inhibition mode of 2-(4-(benzyloxy)-5-(hydroxyl) phenyl) benzothiazole derivatives, dialysis method^37^ was carried out with **3h** as the test compound. Safinamide (reversible MAO-B inhibitor) and rasagiline (irreversible MAO-B inhibitor) were used as control compounds. According to the literature^38^, the MAO-B inhibitory mode can be divided into three types according to the MAO-B catalytic activities in dialysis groups (reversible inhibition: MAO-B catalytic activity ≥ 80%; irreversible inhibition: MAO-B catalytic activity ≤ 20%; quasi-reversible inhibition: 20% < MAO-B catalytic activity < 80%).

**Figure 3.**
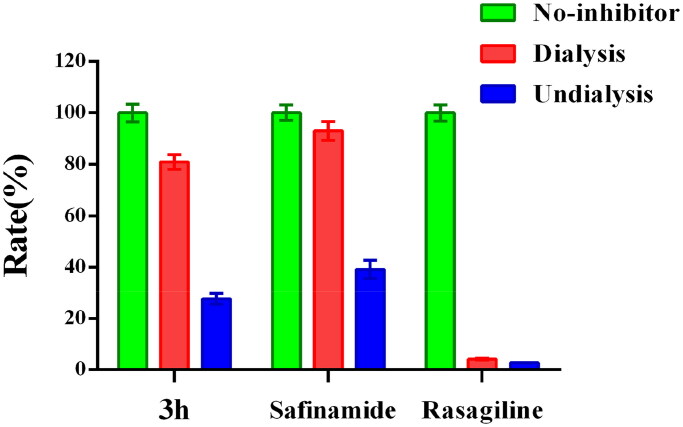
Reversibility of the MAO-B inhibition by **3h**, safinamide, and rasagiline.

The experimental result was shown in Figure 3. Not surprisingly, the MAO-B catalytic activities in the undialysis groups were 27.6% (**3h**), 39.3% (safinamide) and 2.7% (rasagiline). Besides, the MAO-B activities in dialysis group of safinamide recovered to 93.0%, while the MAO-B activities in rasagiline dialysis group only recovered to 4.2%, reflecting their MAO-B inhibitory behaviour were reversible and irreversible, respectively. Compound **3h** exhibited similar MAO-B inhibitory mode with safinamide, and the MAO-B catalytic activities in its dialysis group recovered to 80.9%. It revealed that **3h** is a reversible MAO-B inhibitor.

#### Docking study

**Figure 4.**
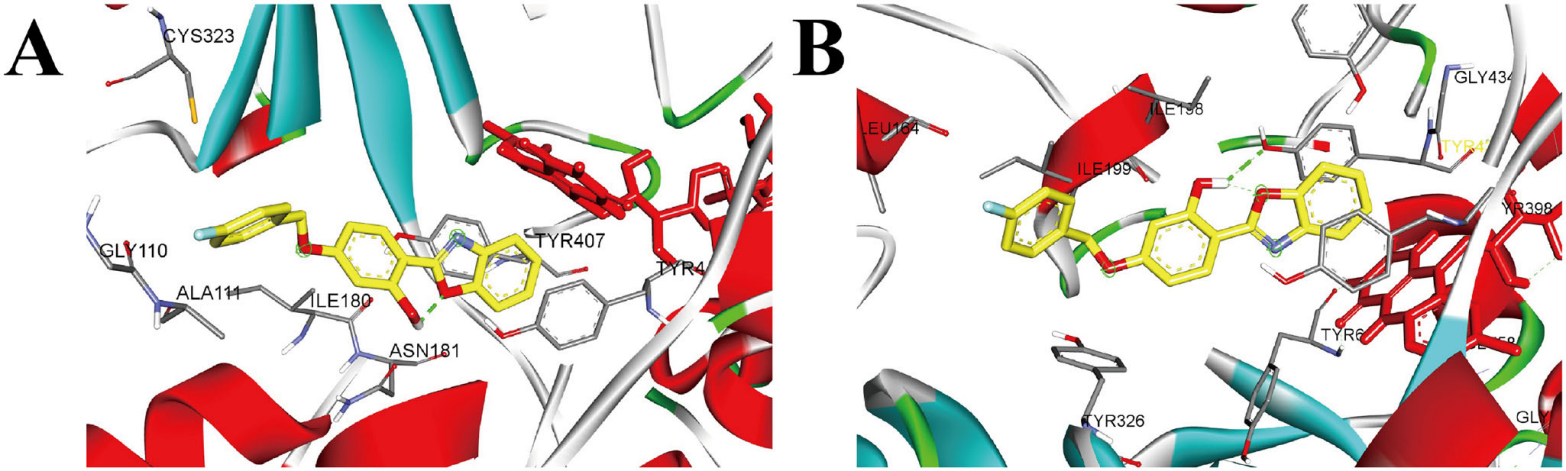
(A) The docking mode of compound **3h** with *h*MAO-A (PDB ID: *2Z5X*), (B) The docking mode of compound **3h** with *h*MAO-B (PDB ID: *2V5Z*), **3h** and amino acid residues that participate in the interactions were displayed as sticks and coloured by the element, hydrogen bonds are showed as green dashed lines.

Molecular docking study^39^ was carried out to clarify the interaction modes of **3h** with MAO-A/B by using the software packages of Autodock 4.2. The crystal structures of *h*MAO-A (PDB ID: *2Z5X*) and *h*MAO-B (PDB ID: *2V5Z*) were used for this study, and the result was depicted in Figure 4. Obviously, representative compound **3h** showed strong interactions with MAO-B. In detail, **3h** induced one hydrogen bond interaction with Tyr435 (**3h**-OH•••O-Tyr435) and formed two parallel π–π interactions with Tyr435 and Tyr398. Besides, **3h** also adopted T-shaped π–π interaction with flavin adenine dinucleotide (FAD), and exhibited hydrophobic interactions with the amino acid residues Leu164, Tyr326, Ile199, Ile198, Tyr188, Tyr60, and Gly434. Therefore, compound **3h** had strong affinity to MAO-B and showed potent MAO-B inhibition.

As for the binding mode of **3h** with MAO-A (Figure 4(A)), **3h** formed parallel π–π interaction with Tyr444 and adopted T-shaped π–π interaction with FAD. Additionally, **3h** also displayed hydrophobic interactions with the amino acid residues Cys323, Gly110, Ala111, Ile180, Asn181 and Tyr407, while no hydrogen bond interaction was observed in the docking complex of **3h**-MAO-A. Therefore, **3h** exhibited relatively weak inhibition on MAO-A, and displayed selective inhibition on MAO-B.

#### *In vitro* antioxidant activity study

Oxygen radical absorbance capacity assay which used fluorescein (ORAC-FL) was performed to measure the antioxidant activities of 2-(4-(benzyloxy)-5-(hydroxyl) phenyl) benzothiazole derivatives^40^. Trolox (6-hydroxy-2,5,7,8-tetramethyl-chroman-2-carboxylic acid), was used as a standard, and the result was shown in Table 1. Obviously, all the derivatives showed excellent antioxidant activities in this assay, with ORAC-FL values between 2.13 and 2.48 Trolox equivalents. In particular, **3k** exhibited the most potent activity (ORAC-FL value = 2.48-fold of Trolox), and the representative compound **3h** also displayed good antioxidant activity with an ORAC-FL value of 2.14 Trolox equivalents. Compared with compound **3a**, all the other derivatives showed much higher ORAC-FL value, indicating the introduction of substituent groups on benzyloxy ring was favourable for the improvement of radical scavenge activities. Besides, compound **3k** and **3l** showed superior antioxidant activity than other derivatives. It reflected that introduction of alkyl substituents on benzyloxy ring was more beneficial for the improvement of *in vitro* antioxidant activity. However, the position and number of substituents seem to have little influence on the antioxidative activities.

#### Metal-chelating study

**Figure 5.**
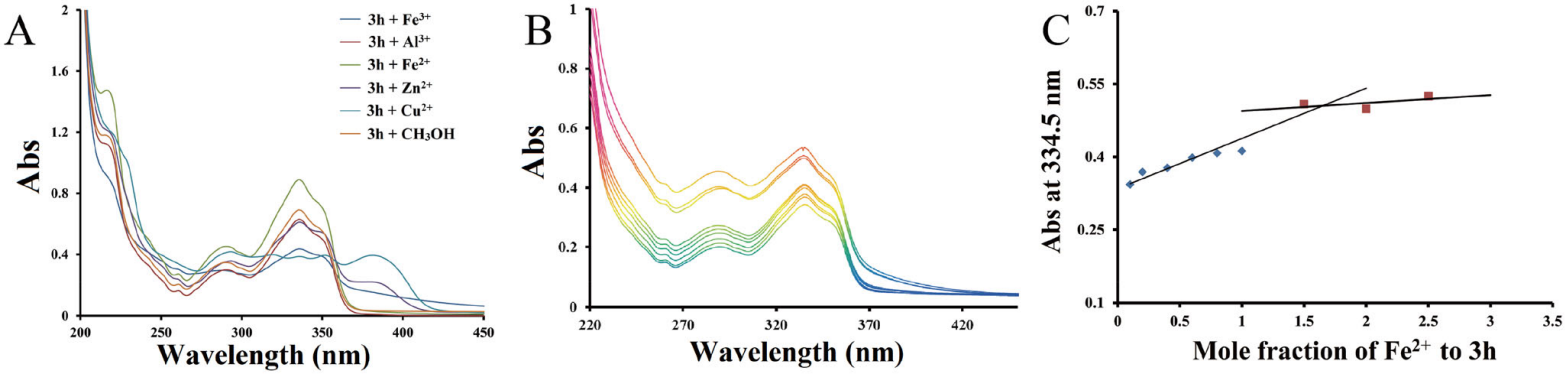
(A) UV spectra of compound **3h** (75.0 μM in methanol) with or without FeSO_4_, ZnCl_2_, CuCl_2_, FeCl_3_, or AlCl_3_ (75.0 μM in methanol); (B) UV spectra of compound **3h** (75.0 μM in methanol) with FeSO_4_ (0, 7.5, 15.0, 30.0, 45.0, 60.0, 75.0, 112.5, 150.0, and 187.5 μM); (C) Determination of the stoichiometry of **3h**-Fe^2+^ complex by using the molar ratio method of titrating the methanol solution of compounds **3h** with ascending amounts of FeSO_4_.

UV spectra method^41^ was performed to measure the metal chelating ability of **3h**, and the result was shown in Figure 5. Obviously, the UV spectra were significantly changed after the addition of Fe^3+^, Al^3+^, Zn^2+^, Cu^2+^, Fe^2+^, reflecting that **3h** could interact with these biometals. Particularly, after the addition of FeSO_4_, maximum increase in absorption was detected. Besides, after the addition of CuCl_2_, maximum absorption peak shifted from 335.5 to 382.0 nm. Therefore, **3h** showed strong interaction with Fe^2+^ and Cu^2+^ and can play the role in removing the excess metals in PD brain.

Because Fe^2+^ played a crucial role in the pathogenesis of PD, the stoichiometry of **3h**-Fe^2+^ complex was further measured by using molar ratio method. **3h** was incubated with various concentrations of FeSO_4_ (0–187.5 µM), and the electronic spectra were detected. As shown in Figure 5(C), the absorbance at 385 nm initially increased linearly and then became plateaued with the increase of Fe^2+^ concentration. The point for the straight lines to intersect was determined to be at a mole fraction of 1.65, revealing an almost 2:3 stoichiometry for **3h**-Fe^2+^ complex.

#### *In vitro* blood-brain barrier permeation study

A parallel artificial membrane permeation assay was performed to predict the blood-brain barrier (BBB) permeation ability of **3h**^42^. To gain the standard, the permeabilities of 11 drugs were measured firstly, and then the obtained results (Table S1) were compared with the values reported by literature. Not surprisingly, good linear correlation was observed: *P_e_* (exp.) = 0.8792 × *P_e_* (bibl.) − 0.0616 (*R*^2^ = 0.9555) (Figure S1). Further considering the limit established by Di et al.^43^, we used 3.46 × 10^−6 ^cm/s as the standard, and compounds which showed better permeabilities than the standard could cross BBB (Table S2). Then the predicted BBB permeability of **3h** was measured, the result shown in Table 2 revealed that the *P_e_* value of **3h** (4.03 × 10^−6 ^cm/s) was higher than the standard, meaning that **3h** could cross the BBB and reach its therapeutic targets.

**Table 2.** Permeability results *P*_e_ (×10^−6 ^cm/s) for **3h** and its predicted penetration into CNS.

Compound^a^	*P*_e_ (×10^−6^ cm/s)^b^	Prediction
**3h**	4.03 ± 0.14	CNS+

^a^**3h** were dissolved in DMSO at 5 mg/mL and diluted with PBS/EtOH (70:30). The final concentration of each compound was 100 μg/mL.

^b^Data are the mean ± *SD* of three independent experiments.

#### *In vitro* neuroprotective effect study

**Figure 6.**
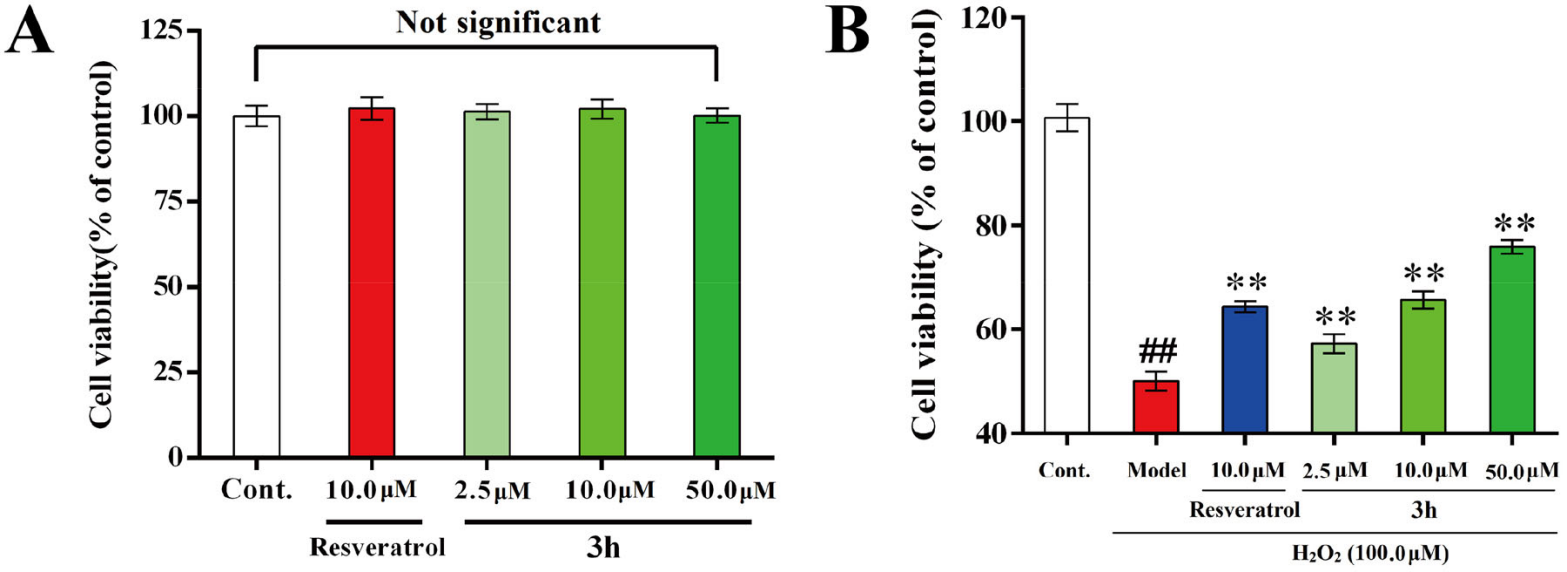
(A) Cytotoxicity of **3h** towards PC-12 cells. (**B**) Neuroprotective effects of **3h** and resveratrol. Three independent experiments were performed in triplicate. Data were expressed as mean ± *SD*. ^##^*p* < 0.01 *vs.* control; **p < 0.01 *vs.* model group.

MTT method was used to detect the *in vitro* neuroprotective effect of **3h** on H_2_O_2_ induced PC-12 cells injury, with resveratrol as positive control^44^. Firstly, the cytotoxicity of **3h** to PC-12 cells was measured. As shown in Figure 6(A), the positive control resveratrol did not significantly alter the PC-12 cell viability at the concentration of 10.0 µM, and **3h** also did not exhibit cytotoxicity up to the concentration of 50.0 µM. Then the neuroprotective effect of **3h** was further tested, and the result was shown in Figure 6(B). Noticeably, the cell viability was significantly reduced to 50.1% of control value when the PC-12 cells were exposed to H_2_O_2_ (100 µM), while treatment with resveratrol (10.0 µM) could reverse the cell viability to 64.3% of control value, indicating resveratrol possesses significant neuroprotective effect. Besides, treatment with **3h** could also increase the cell viability to 57.2, 65.6, and 75.9% of control value at the concentrations of 2.5, 10.0, and 50.0 µM, respectively. Therefore, representative compound **3h** exhibited similar neuroprotective effect with resveratrol. Moreover, the protective potency of **3h** displayed a dose dependent manner, which also verified that **3h** is not toxic towards the PC-12 cells up to the concentration of 50 µM. Overall, these results revealed that **3h** possessed excellent neuroprotective effect because it could reduce the oxidative damage by acting as a scavenger of H_2_O_2_.

#### *In vitro* anti-neuroinflammation study

**Figure 7.**
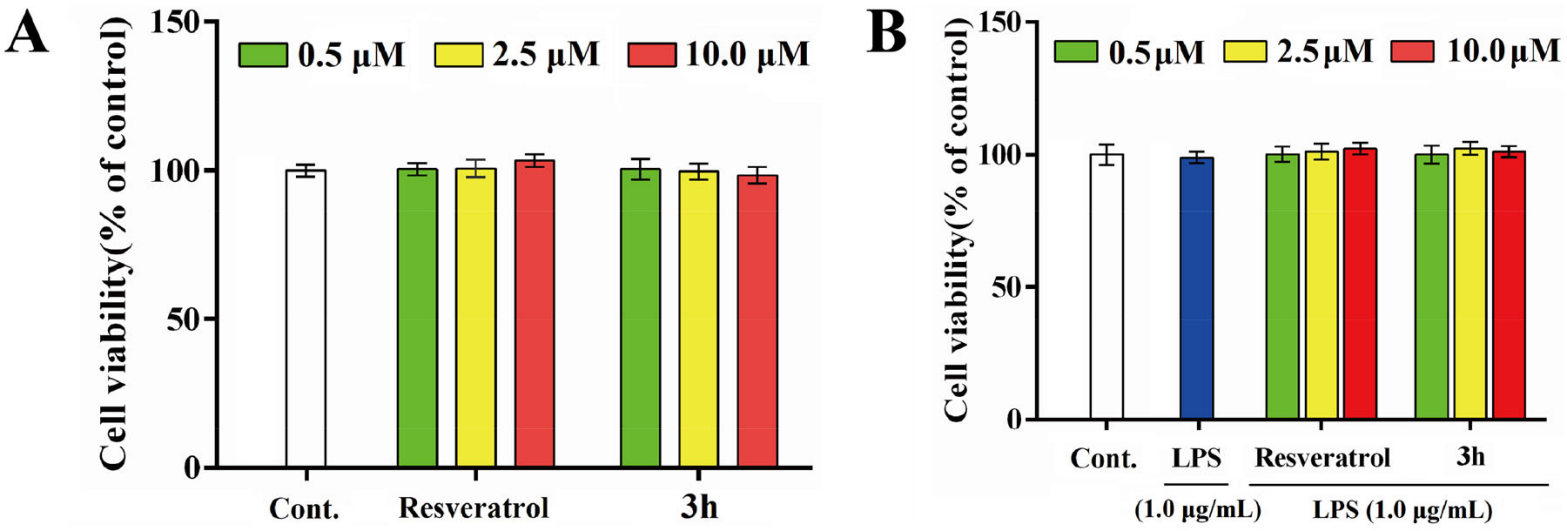
The effects of resveratrol and **3h** on the cell viability of BV-2 cells (A) without LPS and (B) in the presence of LPS (1.0 μg/mL). Each assay was carried out at least three times and data are expressed as mean ± *SD.*

The *in vitro* anti-neuroinflammatory activity of **3h** was detected by using LPS-induced inflammation mode in BV-2 cells, with resveratrol as the positive control compound^45^. The cytotoxicities of resveratrol and **3h** to BV-2 cells were firstly detected by MTT method. As shown in Figure 7, resveratrol and **3h** did not significantly alter the BV-2 cell viability up to the concentration of 10.0 µM in the presence or absence LPS (1.0 µg/mL). It reflected that resveratrol and representative compound **3h** possessed low cytotoxicity to BV-2 cells, and LPS (1.0 µg/mL) also did not influence the cell viability of BV-2 cells.

**Figure 8.**
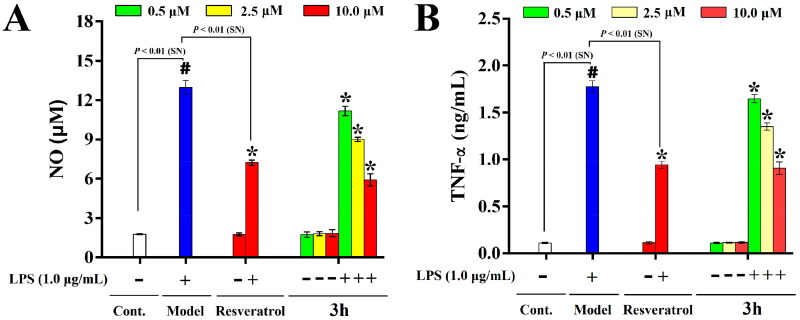
The inhibitory effects of resveratrol and **3h** on NO release (A) and TNF-*α* production (B) in LPS-activated BV-2 microglia cells. The data are expressed as the mean ± *SD* from three independent experiments. ^#^*p* < 0.05 *vs.* control group; *p < 0.05 *vs.* model group.

Then, Griess method was performed to measure the inhibitory effect of **3h** on LPS-induced NO release in BV-2 cells^46^. The result was shown in Figure 8(A). Noticeably, the concentration of NO in control group is comparative low, while it was remarkably increased when BV-2 cells were exposed to LPS (1.0 µg/mL). It reflected that LPS could induce the activation of BV-2 cells and induce neuroinflammation. Besides, treatment of resveratrol (10.0 µM) significantly reduced the LPS-induced NO production, indicating resveratrol have remarkable anti-inflammatory activities (51.2% inhibition at 10.0 µM). Additionally, treatment of **3h** also decreased the NO production, and **3h** exhibited more potent inhibitory effect than resveratrol, and its inhibitory rates were 15.8, 35.8, and 63.7% at the concentration of 0.5, 2.5, and 10.0 µM. Moreover, the effect of **3h** showed a dose dependent manner, which verified that the inhibitory effect of **3h** is not due to the cytotoxicity.

ELISA method was carried out to detect the inhibitory effects of **3h** on LPS-induced TNF-α production^47^. As shown in Figure 8(B), the level of TNF-*α* expression was dramatically raised when BV-2 cells were exposed to LPS (1.0 µg/mL). Besides, the treatment of resveratrol also significantly decreased the production of TNF-*α*, and its inhibitory rate was 50.3% at the concentration of 10.0 µM. Similarly, the inhibitory effect of **3h** was more potent than resveratrol, with inhibition rates of 7.8, 25.7, and 52.5% at the concentration of 0.5, 2.5, and 10.0 µM, and the effect of **3h** also showed a dose dependent manner. These results revealed that representative compound **3h** possessed low cytotoxicity on BV-2 cells and good anti-neuroinflammatory activity *in vitro*.

#### Study of inhibitory effect on LPS-induced ROS release *in vitro*

**Figure 9.**
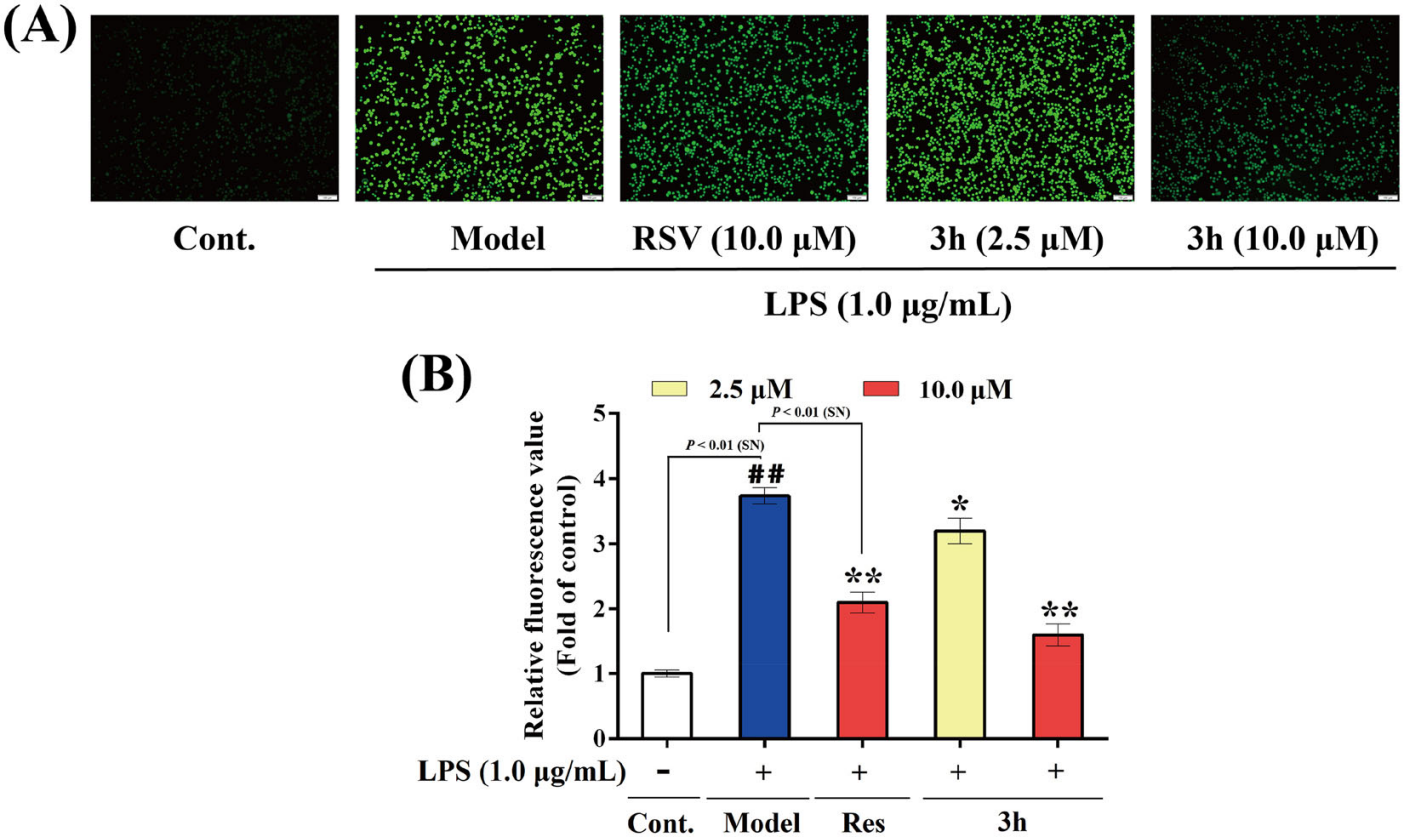
The inhibitory effects of resveratrol and **3h** on LPS-induced ROS release in BV-2 microglia cells. (A) representative images, (B) relative fluorescence values obtained by microplate reader. The data are expressed as the mean ± *SD* from three independent experiments. ^##^*p* < 0.01 *vs.* control; *p < 0.05 *vs.* model group; **p < 0.01 *vs.* model group.

The inhibitory effect of **3h** on LPS-induced ROS release in BV-2 cells *in vitro* was detected by using 2′,7′-dichlorofluorescein diacetate (DCFH-DA) as probe to quantify the ROS level in LPS-treated BV-2 cells^48^. The result was shown in Figure 9. Noticeably, the fluorescence intensity of control group was relatively low, indicating low level of ROS. However, when the BV-2 cells were treated with LPS (1.0 µg/mL), the fluorescence intensity dramatically increased. It revealed that LPS (1.0 µg/mL) could trigger the release of ROS in BV-2 cells. Besides, the treatment of resveratrol and **3h** significantly reduced the fluorescence intensity, so both resveratrol and **3h** have the ability to relieve the rise in oxidative stress level induced by LPS. The fluorescence values were further detected by using microplate reader (Figure 9(B)), and then the inhibition rates were calculated. The inhibition rate of resveratrol was 59.8%, while the inhibition rates of **3h** were 19.8 and 78.0% at the concentration of 2.5 and 10.0 µM. Therefore, **3h** showed slightly more potent inhibitory effect than resveratrol on LPS-induced ROS release *in vitro*.

## Conclusion

A series of 2-(4-(benzyloxy)-5-(hydroxyl) phenyl) benzothiazole derivatives was designed, synthesised and evaluated as multi-functional anti-PD agents. The biological screening indicated that most derivatives possessed potent MAO-B inhibitory activities and anti-oxidative abilities. Among them, compound **3h** raised particular interest, because **3h** showed the most potent MAO-B inhibitory activity (IC_50_ = 0.062 µM) and high antioxidant activity (ORAC = 2.27 Trolox equivalent). Further kinetic study and reversibility study revealed that its MAO-B inhibitory mode was competitive and reversible. Additionally, the molecular study disclosed the interaction modes of **3h** with MAO-A and MAO-B. Besides, **3h** also exhibited good metal chelating ability, and can form **3h**-Fe^2+^ complex with an almost 2:3 stoichiometry. Furthermore, **3h** displayed appropriate BBB permeability and significant neuroprotective effect. Moreover, *in vitro* anti-neuroinflammation study showed that **3h** had the ability to inhibit the release of NO, TNF-*α* and ROS in LPS-treated BV-2 cells. Overall, these results revealed that **3h** was a promising anti-PD agent, which is worthy of being further studied.

## Experimental section

### Chemistry

All reagents were purchased from commercial suppliers and were used without further purification. The reactions were monitored by thin-layer chromatography (TLC), and the spots in TCL were detected under UV light (254 or 365 nm). Column chromatography was carried out using silica gel (230–400 mesh) purchased from General-Reagent (China). The ^1^H NMR and ^13^C NMR spectra were recorded in CDCl_3_ or DMSO-*d*_6_ on a Varian INOVA spectrometer at 25 °C with TMS as the internal standard. The mass spectra of target compounds were detected by the Shimadzu LC-MS 2020 Spectrometer. The purity of target compound was measured on a Shimadzu LC-10Avp plus system by using a Diamonsil C18 column (4.6 × 150 mm, 5 µM).

#### The synthesis of intermediate 2^31^

To a solution of 2-aminothiophenol (0.43 ml, 3.98 mmol) and 2,4-dihydroxybenzaldehyde (605 mg, 4.38 mmol) in DMF (15 ml), Na_2_S_2_O_5_ (1.13 g, 5.94 mmol) was added. After addition, the mixture was refluxed under nitrogen atmosphere for 2 h. After the reaction was completed, the reaction mixture was allowed to cool and H_2_O (30 ml) was poured into it. The mixture was filtered, and the filter cake was collected and dried *in vacuo* to obtain crude intermediate **2**, which was further purified by recrystallization from ethanol to afford the pure product as brown solid, yield 65.7%, mp > 220 °C. ^1^H NMR (400 MHz, DMSO-*d_6_*) *δ* 11.68 (brs, 1H), 10.20 (brs, 1H), 8.09 (d, *J* = 8.0 Hz, 1H), 7.98–7.92 (m, 2H), 7.50 (t, *J* = 7.2 Hz, 1H), 7.39 (t, *J* = 7.2 Hz, 1H), 6.46 (brs, 2H).

#### General procedure for the synthesis of 3a ∼ o^32^

Intermediate **2** (36.5 mg, 0.15 mmol), substituted benzyl chloride or benzyl bromide (0.16 mmol), NaHCO_3_ (18.9 mg, 0.23 mmol), acetonitrile (5.0 ml) and KI (catalytic amount) were added to a round bottom flask (10.0 ml) successively. The suspension was heated to 60 °C under a nitrogen atmosphere for 30 h. Then, the suspension was filtrated, and the filtrate was evaporated under reduced pressure. Subsequently, the residue was purified by column chromatography to afford corresponding target compounds.

##### 2-(Benzo[*d*]thiazol-2-yl)-5-(benzyloxy)phenol (3a)

**3a** was obtained as brown solid, mp 131.5–132.9 °C. ^1^H NMR (400 MHz, DMSO-*d*_6_) *δ* 11.86 (s, 1H), 8.10 (d, *J* = 7.6 Hz, 1H), 8.03 (d, *J* = 8.4 Hz, 1H), 8.00 (d, *J* = 8.4 Hz, 1H), 7.53–7.35 (m, 7H), 6.70 (d, *J* = 8.4 Hz, 1H), 6.69 (s, 1H), 5.18 (s, 2H). ^13^C NMR (100 MHz, DMSO-*d*_6_) *δ* 166.3, 162.3, 158.6, 151.1, 137.0, 133.9, 130.4, 129.0 (2 C), 128.5, 128.2 (2 C), 126.9, 125.2, 122.4, 122.1, 112.1, 108.3, 102.6, 69.9. ESI-MS *m/z*: 332.1 [M − H]^−^.

##### 2-(Benzo[*d*]thiazol-2-yl)-5-((2-fluorobenzyl)oxy)phenol (3b)

**3b** was obtained as brown solid, mp 107.4–108.6 °C. ^1^H NMR (400 MHz, CDCl_3_) *δ* 12.75 (brs, 1H), 7.93 (d, *J* = 8.0 Hz, 1H), 7.86 (d, *J* = 8.0 Hz, 1H), 7.58 (d, *J* = 8.4 Hz, 1H), 7.52–7.46 (m, 2H), 7.38–7.32 (m, 2H), 7.18 (t, *J* = 7.2 Hz, 1H), 7.11 (t, *J* = 8.8 Hz, 1H), 6.69 (s, 1H), 6.60 (d, *J* = 8.0 Hz, 1H), 5.18 (s, 2H). ^13^C NMR (100 MHz, CDCl_3_) *δ* 169.2, 162.3, 161.7–159.3 (d, *J*_C-F_ = 245.8 Hz), 159.9, 151.8, 132.2, 130.0–129.9 (d, *J*_C-F_ = 8.2 Hz), 129.8, 129.7, 126.6, 125.1, 124.4–124.3 (d, *J*_C-F_ = 3.5 Hz), 123.6–123.5 (d, *J*_C-F_ = 14.1 Hz), 121.7, 121.4, 115.6–115.4 (d, *J*_C-F_ = 20.8 Hz), 110.8, 108.1, 102.4, 63.9–63.8 (d, *J*_C-F_ = 4.4 Hz). ESI-MS *m/z*: 350.0 [M − H]^−^.

##### 2-(Benzo[*d*]thiazol-2-yl)-5-((2-chlorobenzyl)oxy)phenol (3c)

**3c** was obtained as brown solid, mp 113.9–135.1 °C. ^1^H NMR (400 MHz, DMSO-*d_6_*) *δ* 11.87 (brs, 1H), 8.11 (d, *J* = 8.0 Hz, 1H), 8.08 (d, *J* = 8.4 Hz, 1H), 8.01 (d, *J* = 8.0 Hz, 1H), 7.63–7.61 (m, 1H), 7.56–7.50 (m, 2H), 7.45–7.40 (m, 3H), 6.75 (d, *J* = 2.4 Hz, 1H), 6.73–6.71 (m, 1H), 5.24 (s, 2H). ^13^C NMR (100 MHz, DMSO-*d_6_*) *δ* 166.1, 162.1, 158.5, 151.9, 134.3, 134.0, 133.1, 130.6, 130.5, 130.4, 129.9, 127.9, 126.9, 125.2, 122.4, 122.1, 112.4, 108.2, 102.5, 87.5. ESI-MS *m/z*: 366.0 [M − H]^−^.

##### 2-(Benzo[*d*]thiazol-2-yl)-5-((3-fluorobenzyl)oxy)phenol (3d)

**3d** was obtained as brown solid, mp 101.7–102.3 °C. ^1^H NMR (400 MHz, CDCl_3_) *δ* 12.77 (brs, 1H), 7.94 (d, *J* = 8.0 Hz, 1H), 7.87 (d, *J* = 8.0 Hz, 1H), 7.59 (d, *J* = 8.8 Hz, 1H), 7.48 (td, *J*_1_ = 8.0 Hz, *J*_2_ = 1.2 Hz, 1H), 7.38 (t, *J* = 8.0 Hz, 1H), 7.35 (d, *J* = 8.0 Hz, 1H), 7.22–7.16 (m, 2H), 7.04 (td, *J*_1_ = 8.4 Hz, *J*_2_ = 1.6 Hz, 1H), 6.65 (d, *J* = 2.4 Hz, 1H), 6.60 (dd, *J*_1_ = 8.4 Hz, *J*_2_ = 2.4 Hz, 1H), 5.11 (s, 2H). ^13^C NMR (100 MHz, CDCl_3_) *δ* 169.2, 164.2–161.8 (d, *J*_C-F_ = 244.9 Hz), 162.2, 159.9, 151.8, 138.9 (d, *J*_C-F_ = 7.3 Hz), 132.2, 130.3–130.2 (d, *J*_C-F_ = 8.2 Hz), 129.7, 126.6, 125.1, 122.8 (d, *J*_C-F_ = 3.0 Hz), 121.7, 121.4, 115.2–115.0 (d, *J*_C-F_ = 21.0 Hz), 114.4–114.2 (d, *J*_C-F_ = 22.0 Hz), 110.8, 108.2, 102.4, 69.3 (d, *J*_C-F_ = 1.9 Hz). ESI-MS *m/z*: 350.1 [M − H]^−^.

##### 2-(Benzo[*d*]thiazol-2-yl)-5-((3-chlorobenzyl)oxy)phenol (3e)

**3e** was obtained as brown solid, mp 123.0–123.8 °C. ^1^H NMR (400 MHz, DMSO-*d_6_*) *δ* 11.89 (brs, 1H), 8.10 (d, *J* = 7.6 Hz, 1H), 8.03 (d, *J* = 8.8 Hz, 1H), 8.00 (d, *J* = 8.0 Hz, 1H), 7.54–7.49 (m, 2H), 7.45–7.39 (m, 4H), 6.73–6.68 (m, 2H), 5.20 (s, 2H). ^13^C NMR (100 MHz, DMSO-*d_6_*) *δ* 166.3, 162.1, 158.6, 151.9, 139.7, 133.9, 133.6, 130.9, 130.4, 128.4, 127.8, 126.9, 126.7, 125.3, 122.4, 122.1, 112.2, 108.3, 102.7, 69.0. ESI-MS *m/z*: 366.0 [M − H]^−^.

##### 2-(Benzo[*d*]thiazol-2-yl)-5-((3-(trifluoromethyl)benzyl)oxy)phenol (3f)

**3f** was obtained as brown solid, mp 118.4–119.7 °C. ^1^H NMR (400 MHz, CDCl_3_) *δ* 12.78 (brs, 1H), 7.94 (d, *J* = 8.4 Hz, 1H), 7.87 (d, *J* = 8.0 Hz, 1H), 7.72 (s, 1H), 7.65–7.59 (m, 3H), 7.53 (t, *J* = 8.0 Hz, 1H), 7.48 (t, *J* = 8.0 Hz, 1H), 7.38 (t, *J* = 8.0 Hz, 1H), 6.67 (d, *J* = 2.4 Hz, 1H), 6.61 (dd, *J*_1_ = 8.8 Hz, *J*_2_ = 2.4 Hz, 1H), 5.16 (s, 2H). ^13^C NMR (100 MHz, CDCl_3_) *δ* 169.1, 162.1, 159.9, 151.8, 137.4, 132.2, 131.3–130.9 (d, *J*_C-F_ = 32.2 Hz), 130.6, 129.8, 129.2, 126.6, 125.4–122.7 (d, *J*_C-F_ = 270.7 Hz), 125.1, 125.1–124.9 (q, *J*_C-F_ = 3.7 Hz), 124.2–124.1 (q, *J*_C-F_ = 3.7 Hz), 121.8, 121.4, 110.9, 108.1, 102.4, 69.3. ESI-MS *m/z*: 400.1 [M − H]^−^.

##### 2-(Benzo[*d*]thiazol-2-yl)-5-((3-methoxybenzyl)oxy)phenol (3g)

**3g** was obtained as brown solid, mp 90.5–92.1 °C. ^1^H NMR (400 MHz, CDCl_3_) *δ* 12.77 (brs, 1H), 7.93 (d, *J* = 7.6 Hz, 1H), 7.86 (d, *J* = 8.0 Hz, 1H), 7.58 (d, *J* = 8.4 Hz, 1H), 7.48 (td, *J*_1_ = 8.0 Hz, *J*_2_ = 1.2 Hz, 1H), 7.37 (td, *J*_1_ = 8.0 Hz, *J*_2_ = 0.8 Hz, 1H), 7.32 (t, *J* = 8.0 Hz, 1H), 7.02 (d, *J* = 7.6 Hz, 1H), 6.99 (brs, 1H), 6.88 (dd, *J*_1_ = 8.0 Hz, *J*_2_ = 2.4 Hz, 1H), 6.67 (d, *J* = 2.4 Hz, 1H), 6.60 (dd, *J*_1_ = 8.8 Hz, *J*_2_ = 2.4 Hz, 1H), 5.09 (s, 2H), 3.83 (s, 3H). ^13^C NMR (100 MHz, CDCl_3_) *δ* 169.2, 162.5, 159.9, 151.9, 137.9, 132.2, 129.9, 129.8, 129.7, 126.6, 125.1, 121.7, 121.4, 119.7, 113.7, 112.9, 110.6, 108.2, 102.4, 70.0, 55.3. ESI-MS *m/z*: 362.1 [M − H]^−^.

##### 2-(Benzo[*d*]thiazol-2-yl)-5-((4-fluorobenzyl)oxy)phenol (3h)

**3h** was obtained as brown solid, mp 135.1–136.5 °C. ^1^H NMR (400 MHz, CDCl_3_) *δ* 12.79 (brs, 1H), 7.93 (d, *J* = 8.4 Hz, 1H), 7.86 (d, *J* = 8.0 Hz, 1H), 7.58 (d, *J* = 8.8 Hz, 1H), 7.48 (dd, *J*_1_ = 8.0 Hz, *J*_2_ = 1.2 Hz, 1H), 7.44–7.40 (m, 2H), 7.37 (td, *J*_1_ = 8.0 Hz, *J*_2_ = 0.8 Hz, 1H), 7.12–7.06 (m, 2H), 6.66 (d, *J*_2_ = 2.8 Hz, 1H), 6.58 (dd, *J*_1_ = 8.8 Hz, *J*_2_ = 2.8 Hz, 1H), 5.06 (s, 2H). ^13^C NMR (100 MHz, CDCl_3_) *δ* 169.2, 163.9–161.4 (d, *J*_C-F_ = 245.0 Hz), 162.3, 159.9, 151.8, 132.2, 132.1–132.0 (d, *J*_C-F_ = 3.2 Hz), 129.7, 129.5–129.4 (d, *J*_C-F_ = 8.3 Hz, 2 C), 126.6, 125.1, 121.7, 121.4, 115.7–115.5 (d, *J*_C-F_ = 21.4 Hz, 2 C), 110.7, 108.2, 102.3, 69.5. ESI-MS *m/z*: 350.0 [M − H]^−^.

##### 2-(Benzo[*d*]thiazol-2-yl)-5-((4-chlorobenzyl)oxy)phenol (3i)

**3i** was obtained as brown solid, mp 132.8–133.3 °C. ^1^H NMR (400 MHz, DMSO-*d*_6_) *δ* 11.90 (brs, 1H), 8.11 (d, *J* = 8.0 Hz, 1H), 8.05 (d, *J* = 8.0 Hz, 1H), 8.01 (d, *J* = 8.0 Hz, 1H), 7.54–7.47 (m, 5H), 7.42 (td, *J*_1_ = 8.0 Hz, *J*_2_ = 0.8 Hz, 1H), 6.72 (d, *J*_2_ = 2.4 Hz, 1H), 6.69 (dd, *J*_1_ = 8.0 Hz, *J*_2_ = 2.4 Hz, 1H), 5.18 (s, 2H). ^13^C NMR (100 MHz, DMSO-*d*_6_) *δ* 166.2, 162.1, 158.6, 151.9, 136.1, 133.9, 133.0, 130.4, 130.1 (2 C), 129.0 (2 C), 126.9, 125.2, 122.4, 122.1, 112.2, 108.3, 102.6, 69.1. ESI-MS *m/z*: 366.0 [M − H]^−^.

##### 2-(Benzo[*d*]thiazol-2-yl)-5-((4-methylbenzyl)oxy)phenol (3j)

**3j** was obtained as brown solid, mp 145.5–146.2 °C. ^1^H NMR (400 MHz, CDCl_3_) *δ* 12.74 (brs, 1H), 7.93 (d, *J* = 8.0 Hz, 1H), 7.87 (d, *J* = 8.0 Hz, 1H), 7.58 (d, *J* = 8.0 Hz, 1H), 7.48 (t, *J* = 7.2 Hz, 1H), 7.37 (t, *J* = 8.0 Hz, 1H), 7.34 (d, *J* = 8.0 Hz, 2H), 7.21 (d, *J* = 8.0 Hz, 2H), 6.67 (t, *J* = 2.4 Hz, 1H), 6.69 (dd, *J*_1_ = 8.8 Hz, *J*_2_ = 2.4 Hz, 1H), 5.07 (s, 2H), 2.37 (s, 3H). ^13^C NMR (100 MHz, CDCl_3_) *δ* 169.3, 162.6, 159.9, 151.9, 138.1, 133.2, 132.2, 129.7, 129.4 (2 C), 127.7 (2 C), 126.6, 125.1, 121.7, 121.4, 110.5, 108.3, 102.3, 70.1, 21.3. ESI-MS *m/z*: 346.1 [M − H]^−^.

##### 2-(Benzo[*d*]thiazol-2-yl)-5-((4-(tert-butyl)benzyl)oxy)phenol (3k)

**3k** was obtained as brown solid, mp 151.4–152.0 °C. ^1^H NMR (400 MHz, CDCl_3_) *δ* 12.73 (brs, 1H), 7.93 (d, *J* = 8.0 Hz, 1H), 7.86 (d, *J* = 8.0 Hz, 1H), 7.58 (d, *J* = 8.8 Hz, 1H), 7.50–7.35 (m, 6H), 6.68 (d, *J* = 2.4 Hz, 1H), 6.60 (dd, *J*_1_ = 8.8 Hz, *J*_2_ = 2.4 Hz, 1H), 5.08 (s, 2H), 1.33 (s, 9H). ^13^C NMR (100 MHz, CDCl_3_) *δ* 169.3, 162.7, 159.9, 151.9, 151.3, 133.3, 132.2, 129.6, 127.5 (2 C), 126.6, 125.6 (2 C), 125.0, 121.7, 121.4, 110.6, 108.3, 102.3, 70.1, 34.6, 31.3 (3 C). ESI-MS *m/z*: 388.1 [M − H]^−^.

##### 2-(Benzo[*d*]thiazol-2-yl)-5-((4-nitrobenzyl)oxy)phenol (3l)

**3l** was obtained as brown solid, mp 128.5–129.1 °C. ^1^H NMR (400 MHz, DMSO-*d*_6_) *δ* 8.25 (d, *J* = 8.4 Hz, 1H), 8.08 (d, *J* = 8.0 Hz, 1H), 8.03 (d, *J* = 8.4 Hz, 1H), 7.98 (d, *J* = 8.4 Hz, 1H), 7.72 (d, *J* = 8.4 Hz, 2H), 7.50 (t, *J* = 7.6 Hz, 1H), 7.39 (t, *J* = 7.6 Hz, 1H), 6.70 (m, 2H), 5.34 (s, 2H). ^13^C NMR (100 MHz, DMSO-*d*_6_) *δ* 166.2, 161.8, 151.9, 147.5, 145.1, 134.1, 130.3, 128.7 (2 C), 126.8, 125.1, 124.1 (2 C), 122.3, 122.0, 112.6, 107.7, 102.8, 68.7, 29.5. ESI-MS *m/z*: 377.1 [M − H]^−^.

##### 2-(Benzo[*d*]thiazol-2-yl)-5-((3,4-difluorobenzyl)oxy)phenol (3m)

**3m** was obtained as brown solid, mp 136.6–137.8 °C. ^1^H NMR (400 MHz, DMSO-*d_6_*) *δ* 11.90 (brs, 1H), 8.11 (d, *J* = 8.0 Hz, 1H), 8.05 (d, *J* = 8.8 Hz, 1H), 8.01 (d, *J* = 8.0 Hz, 1H), 7.60–7.40 (m, 4H), 7.35 (brs, 1H), 6.72–6.69 (m, 2H), 6.17 (s, 2H). ^13^C NMR (100 MHz, DMSO-*d_6_*) *δ* 166.3, 162.0, 158.6, 151.9, 151.1–148.6 (dd, *J*_C-F_ = 244.0 Hz, *J*_C-F_ = 12.5 Hz), 150.8–148.3 (dd, *J*_C-F_ = 244.2 Hz, *J*_C-F_ = 12.1 Hz), 134.9–134.8 (q, *J*_C-F_ = 3.0 Hz), 133.9, 130.4, 126.9, 125.3, 125.2–125.1 (q, *J*_C-F_ = 3.0 Hz), 122.4, 122.1, 118.2–118.0 (d, *J*_C-F_ = 17.0 Hz), 117.5–117.3 (d, *J*_C-F_ = 17.0 Hz), 112.3, 108.3, 102.7, 68.6. ESI-MS *m/z*: 368.0 [M − H]^−^.

##### 2-(Benzo[*d*]thiazol-2-yl)-5-((3,4-dichlorobenzyl)oxy)phenol (3n)

**3n** was obtained as brown solid, mp 125.3–126.4 °C. ^1^H NMR (400 MHz, DMSO-*d_6_*) *δ* 8.09 (d, *J* = 8.0 Hz, 1H), 8.05 (d, *J* = 9.2 Hz, 1H), 7.99 (d, *J* = 8.0 Hz, 1H), 7.74 (s, 1H), 7.67 (d, *J* = 8.4 Hz, 1H), 7.51 (t, *J* = 7.6 Hz, 1H), 7.45 (d, *J* = 8.4 Hz, 1H), 7.40 (t, *J* = 7.6 Hz, 1H), 6.68 (brs, 2H), 5.18 (s, 2H). ^13^C NMR (100 MHz, DMSO-*d_6_*) *δ* 168.1, 161.9, 160.3, 152.0, 138.5, 134.2, 131.6, 131.2, 130.9, 130.1, 130.0, 128.3, 126.7, 124.8, 122.3, 121.9, 112.8, 107.1, 102.8, 68.2. ESI-MS *m/z*: 340.0 [M − H]^−^.

##### 2-(Benzo[*d*]thiazol-2-yl)-5-((3-chloro-4-fluorobenzyl)oxy)phenol (3o)

**3o** was obtained as brown solid, mp 130.9–131.5 °C. ^1^H NMR (400 MHz, DMSO-*d_6_*) *δ* 11.89 (brs, 1H), 8.10 (d, *J* = 7.6 Hz, 1H), 8.04 (d, *J* = 8.4 Hz, 1H), 8.00 (d, *J* = 8.0 Hz, 1H), 7.71 (d, *J* = 6.8 Hz,1H), 7.53–7.46 (m, 3H), 7.41 (t, *J* = 7.6 Hz, 1H), 7.72–6.69 (m, 2H), 5.16 (s, 2H). ^13^C NMR (100 MHz, DMSO-*d_6_*) *δ* 166.3, 162.0, 158.6, 158.5–156.1 (d, *J*_C-F_ = 245.4 Hz), 151.9, 135.1–135.0 (d, *J*_C-F_ = 4.0 Hz), 133.9, 130.4 (2 C), 129.1–129.0 (d, *J*_C-F_ = 7.7 Hz), 126.9, 125.3, 122.4, 122.1, 120.1–119.9 (d, *J*_C-F_ = 17.7 Hz), 117.6–117.4 (d, *J*_C-F_ = 20.8 Hz), 112.3, 108.3, 102.7, 68.5. ESI-MS *m/z*: 384.0 [M − H]^−^.

### Pharmacology

#### Inhibition experiments of MAOs^33^

Test compound solution (10 µL), MAO-A or MAO-B solution (30 µL, 12.5 µg/mL in PBS) were added to a black 96-wells plate successively. The mixture was incubated at 37 °C for 30 min. Then, kynuramine solution (10 µL, 224 µM for MAO-A or 150 µM for MAO-B in PBS) was added, the mixture was incubated at 37 °C for another 30 min. After incubation, NaOH solution (40 µL, 2 mol/l in water) and H_2_O (100 µL) were added to end the reaction, the fluorescence was measured with excitation and emission wavelengths at 310 and 400 nm on the multifunctional enzyme marker (Thermo Scientific), and the percentage inhibition was calculated by the formula: 100 − (IF_i_ − IF_0_)/(IF_c_ − IF_0_)*100. The IC_50_ was further detected if their percentage inhibition at the concentration of 10 µM was higher than 50%.

#### Kinetic study^35^^,^^36^

The kinetic study of **3h** was conducted by the method which is similar with the inhibition experiment of MAOs. Different concentrations of **3h** (0.124, 0.062, and 0.031 µM) and kynuramine (15, 30, 60, and 90 µM) solutions were selected. The control experiment was performed without **3h**. The method of data analysis has been described previously.

#### Reversibility study^37^

MAO-B solution (0.3 ml, 0.06 mg/mL in PBS containing 5% sucrose) and test compound solution (0.3 ml, 4 × IC_50_) were added to a vial. The mixture was incubated at 37 °C for 30 min and transferred to dialysis bag (MWCO: 10 000, flattening width: 24 mm, length: 8–10 cm). Then, this dialysis bag was placed in 200 ml PBS for 24 h, and the outer PBS was replaced with fresh at 3 and 7 h, respectively. As for the undialysis group, the mixture was placed at 4 °C for 24 h. After dialysis, the mixtures (40 µL) in the dialysis bag were taken out to measure the catalytic activity, and the test method was similar with the inhibition experiment of MAOs. Safinamide was used as positive control and rasagiline was used as negative control. The test compound solution was replaced with PBS buffer at the same volume in the blank group.

#### Molecular docking with MAOs

The molecular docking study was performed by using autodock 4.2 software package^39^. Firstly, the crystal structures of MAO-A (PDB ID: *2z5x*) and MAO-B (PDB ID: *2v5z*) were obtained from PDB website (https://www.rcsb.org/). Then, original ligands (harmine or safinamide) and water molecules were removed from the crystals, and the polar hydrogens were added to the obtained crystals. Subsequently, the chemical structure of **3h** was plotted using chemdraw software, and then the lowest energy conformation of **3h** was calculated in Chem3D and its torsion tree was defined in autodock 4.2 software. After preparation of macromolecules and ligand, the ligand was embed into the macromolecular receptor using autogrid. After the embedment, the autodock program was run, and the flexible docking program generally runs 100 times. The conformation with the highest populated cluster and lowest energy was chosen to the result discussion.

#### *In vitro* antioxidant activity study^40^

Test compounds solution (20 µL, 10 µM) and fluorescein solution (120 µL, 250 nM) were added to a black 96-wells plate in order. The mixture was incubated for 15 min at 37 °C. Then, AAPH solution (60 µL, 40 mM) was added through an automatic sampler. The fluorescence was recorded with excitation and emission wavelengths at 485 and 535 nm on the multifunctional enzyme marker (Thermo Scientific), and the area under the curve (AUC) was calculated automatically. Trolox was used as a reference, and the antioxidant activities of the compounds were expressed as Trolox equivalent. The test compound solution was replaced with PBS (20 µL) in blank well. The antioxidant activities of the test compounds were calculated by the formula: [(AUC_sample_ − AUC_blank_)/(AUC_trolox_ − AUC_blank_)] × [(Trolox concentration/test sample concentration)].

#### Metal-chelating studies^41^

UV spectrum method was conducted to clarify the metal chelating ability of 2-(4-(benzyloxy)-5-(hydroxyl) phenyl) benzothiazole derivatives. Test compound solution (1.0 ml, 75 µM in methanol) and metal ion solution (1.0 ml, 75 µM in methanol) were added to a vial. The vial was placed at room temperature for 2 h. Then, the mixture was removed from the vial to a quartz cell. After transfer, the absorbance of the mixture at 200–800 nm was measured by using UV spectrophotometer (Shimadzu UV-1900i).

As the Fe^2+^ played important role in the pathogenetic process of PD, the stoichiometry of **3h**-Fe^2+^ complex was detected by using molar ratio method. In detail, test compound solution (1.0 ml, 75 µM in methanol) was incubated with Fe^2+^ solution (1.0 ml, 7.5–187.5 µM in methanol) at 37 °C for 30 min. Then, the mixture was removed from the vial to a quartz cell, and the absorbance of mixture at 200–800 nm was measured by using UV spectrophotometer (Shimadzu UV-1900i).

#### *In vitro* blood-brain barrier permeation assay

The method described by Di et al.^43^ was carried out to detect the blood-brain barrier permeation of 2-(4-(benzyloxy)-5-(hydroxyl) phenyl) benzothiazole derivatives. Porcine brain lipid solution (4 µL, 20 mg/mL in n-dodecane) was added dropwise to a filter membrane. Then the membrane was placed still at room temperature for 5 min to form an artificial biomembrane. When this bionics membrane was formed, test compound solution (350 µL, 100 µg/mL) was added to the donor well which was over the artificial biomembrane, and the mixture of PBS and Ethanol (200 µL, 70/30, v/v) was added to receptor well which is under the bionics membrane. Then, the “sandwich” was incubated at 25 °C for 18 h. After incubation, 150 µL solution in both donor well and receptor well were taken out and added to a 96-well quartz cuvette, and the absorbance of the mixture at 200–800 nm was measured. The *P_e_* value was calculated according to the formula depicted by the literature.

#### Study of neuroprotective effect on H_2_O_2_-mediated PC12 cells injury

3-(4,5-dimethylthiazol-2-yl)-2,5-diphenyltetrazolium bromide (MTT) method was conducted to measure the *in vitro* neuroprotective effect of **3h**^44^. Firstly the cytotoxicity of **3h** towards PC-12 cells was detected. In detail, PC-12 cells were seeded into 96-well cell culture plate in DMEM (100 µL, 1 × 10^5^ cells per mL) firstly. Then, these cells were incubated at 37 °C for 24 h. After incubation, test compound solutions (10 µL) were added to the plate, and the mixture was incubated for another 2 h at 37 °C. Then, MTT solution (100 µL, 5 mg/ml in PBS) was added to each well, which would be incubated at 37 °C for final 4 h. After the final incubation, the supernatants were removed, and the formazan was dissolved in DMSO (150 µL). Then, the absorbance at 490 nm was determined by using a Varioskan Flash Multimode Reader (Thermo Scientific). As for the assay to evaluate the neuroprotective effect of **3h**, the procedures were almost similar, but after the incubation of cells with **3h**, H_2_O_2_ (100 µM) was added and the mixture was further incubated for 24 h.

#### Study of anti-neuroinflammatory effect * in vitro*

Firstly, the MTT method was carried out to determine the cytotoxicity of **3h** towards BV-2 cell line^45^. BV-2 cells in the logarithmic phase were seeded into 96-well cell culture plate, followed by the incubation at 37 °C for 24 h at the atmosphere of 5% CO_2_. After incubation, the culture was replaced by fresh one (90 µL without serum), and test compound solution (10 µL) was added. Then, the mixture was incubated for another 30 min, and MTT (100 µL, 0.5 mg/ml in PBS) was added to each well. After the final incubation at 37 °C for 4 h, the supernatants were removed, and the formazan was dissolved in 200 µL of DMSO. Then, the absorbance at 490 nm was determined by using a Varioskan Flash Multimode Reader (Thermo Scientific). As for the method to evaluate the cytotoxicity of **3h** towards LPS-stimulated BV-2 cells, the procedures were almost similar, but after the incubation of cells with **3h**, LPS (10 µL, 1.0 µg/mL) was added, and the mixture was further incubated for 24 h.

ELISA and Griess reaction methods were performed to detect the inhibitory effect of **3h** on the release of TNF-*α* and NO in LPS-stimulated BV-2 cells^46^^,^^47^. BV-2 cells in the logarithmic phase were seeded into 96-well cell culture plate, and these cells were incubated at 37 °C for 24 h at the atmosphere of 5% CO_2_. After incubation, the culture solution was replaced by fresh one (90 µL without serum), and test compound solution (10 µL) was added. After addition, the plate was incubated for 30 min, then LPS (10 µL, 1.0 µg/ml in PBS) was added to each well, and the mixture would be incubated for another 24 h at 37 °C. After the final incubation, the supernatants were taken out to measure the level of TNF-*α* and NO according to the procedures depicted on the kits (kits were purchased from Nanjing Jiancheng Bioengineering Institute).

#### Study of inhibitory effect on LPS-induced ROS release * in vitro^48^*

BV-2 cells were incubated with test compound solution (10 µL) for 30 min, then LPS (10 µL, 1.0 µg/ml in PBS) was added to each well, and each well would be incubated for another 30 min at 37 °C. Then, DCFH-DA (100 µL, 10 µM) was added, and the mixture was incubated for another 40 min at 37 °C. The fluorescence was measured with excitation and emission wavelengths at 488 and 525 nm on an inverted fluorescence microscope.

## Supplementary Material

Supplemental Material
