# Supplementary material for: Discovery of novel 2-(4-(benzyloxy)-5-(hydroxyl) phenyl) benzothiazole derivatives as multifunctional MAO-B inhibitors for the treatment of Parkinson’s disease
Source: J Enzyme Inhib Med Chem. 2023 Feb 2;38(1):2159957. doi: 10.1080/14756366.2022.2159957 (PMC9897792; doi:10.1080/14756366.2022.2159957)
Supplement: Supplemental Material [file IENZ_A_2159957_SM5396.pdf]

Discovery of novel2-(4-(benzyloxy)-5-(hydroxyl) phenyl) benzothiazole derivatives as multifunctional MAO-B inhibitors for the treatment of Parkinson's disease

ZhongchengCao[a], XingyueWang[a], TianlongZhang[a], XianwuFu[a], Fan Zhang[a], Jiang Zhu[b]

[a] *School of Pharmacy, North Sichuan Medical College, Nanchong637000, China.*

[b] *Sichuan Key Laboratory of Medical Imaging, School of Pharmacy and Nanchong Key laboratory of MRI Contrast Agent, North Sichuan Medical College, Nanchong 637000, China.*

Table of Contents

1. Figure S1. Linear correlation between experimental and reported permeability values of commercial drugs, determined using the PAMPA-BBB assay

2. Table S1. Permeability ($P_e \times 10^{-6}$ cm/s) in the PAMPA-BBB assay for 11 commercial drugs used in the experiment validation

3. Table S2. Ranges of permeability of PAMPA-BBB assays ($P_e \times 10^{-6}$ cm/s)

4. The ^{1}H NMR, ^{13}C NMR and ESI-MS spectra of the target compounds

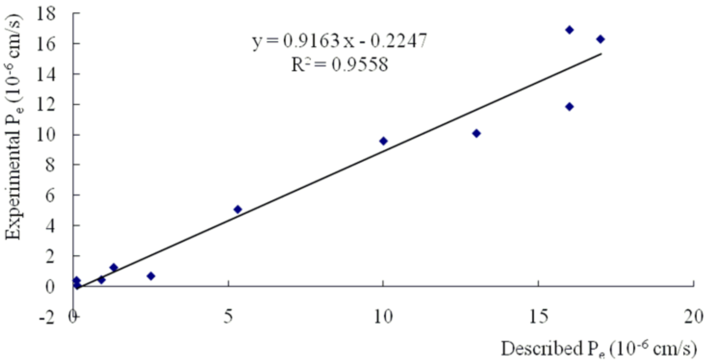

Figure S1. Linear correlation between experimental and reported permeability of commercial drugs using the PAMPA-BBB assay. P_e (exp.) = 0.9163 $\times P_e$ (bibl.) − 0.2247 (R^2 = 0.9558).

Table S1. Permeability P_e ($\times 10^{-6}$ cm/s) in the PAMPA-BBB assay for 11 commercial drugs used in the experiment validation.

Commercial drugs	Bibl[a]	PBS/EtOH (70:30)[b]
Verapamil	16	16.90 ± 0.36
Oxazepam	10	9.60 ± 0.21
Diazepam	16	11.86 ± 0.23
Clonidine	5.3	5.10 ± 0.16
Imipramine	13	10.10 ± 0.22
Testosterone	17	16.30 ± 0.25
Caffeine	1.3	1.28 ± 0.05
Enoxacine	0.9	0.47 ± 0.01
Piroxicam	2.5	0.72 ± 0.02
Norfloxacin	0.1	0.42 ± 0.01
Theophylline	0.12	0.10 ± 0.003

[a]Taken from Ref.[1]
[b]Data are the mean ± SD of three independent experiments.

Table S2. Ranges of permeability of PAMPA-BBB assays ($P_e \times 10^{-6}$ cm/s).

High BBB permeation predicted (CNS +)	P_e> 3.44
Uncertain BBB permeation (CNS +/-)	3.44>P_e> 1.61
Low BBB permeation predicted (CNS -)	P_e<1.61

References

1. Di, L.; Kerns, E. H.; Fan, K.; Mcconnell, O. J.; Carter, G. T. *Eur. J. Med. Chem.***2003**, *38*, 223

4. The ^{1}H NMR, ^{13}C NMR and ESI-MS spectra of the target compounds

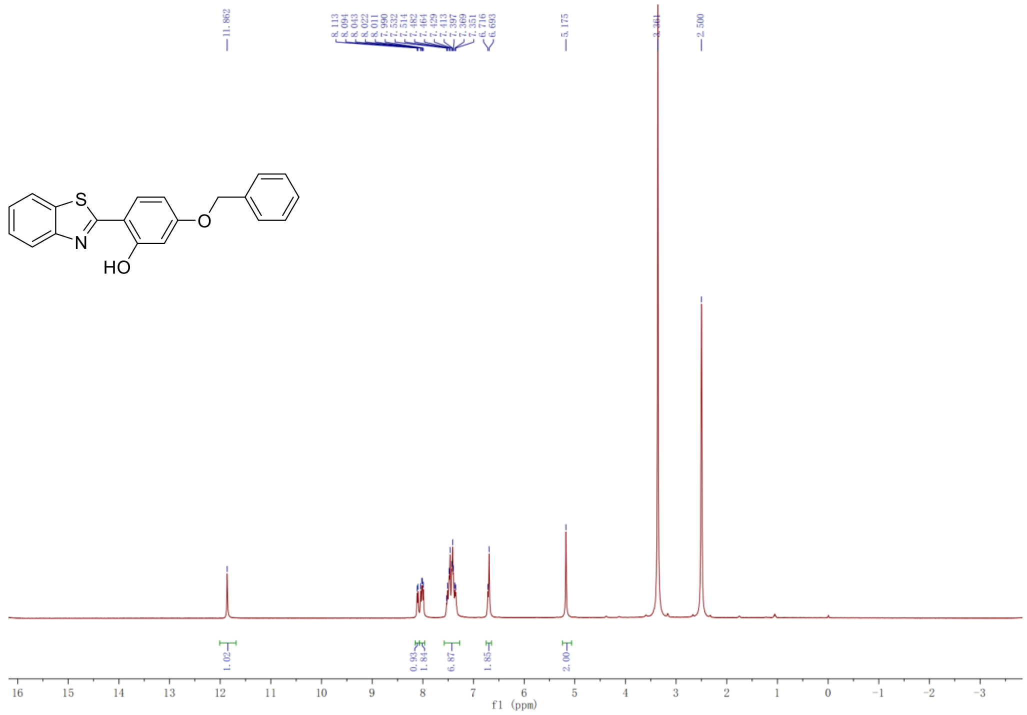

^{1}H NMR of compound 3a (400 MHz, DMSO-d_6)

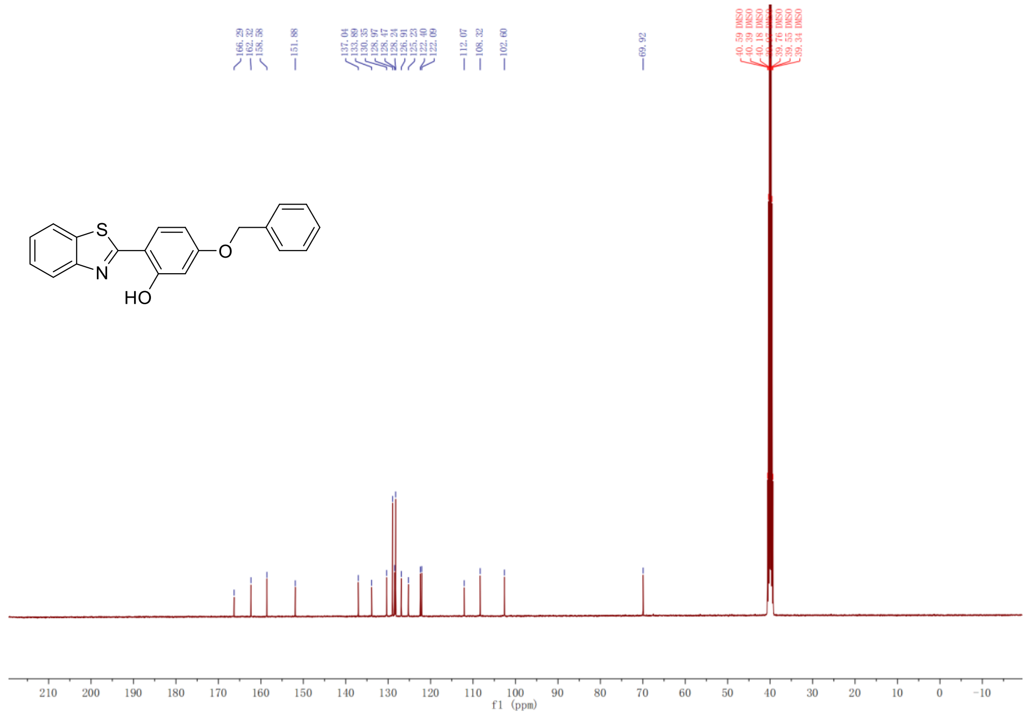

^{13}C NMR of compound 3a (100 MHz, DMSO-d_6)

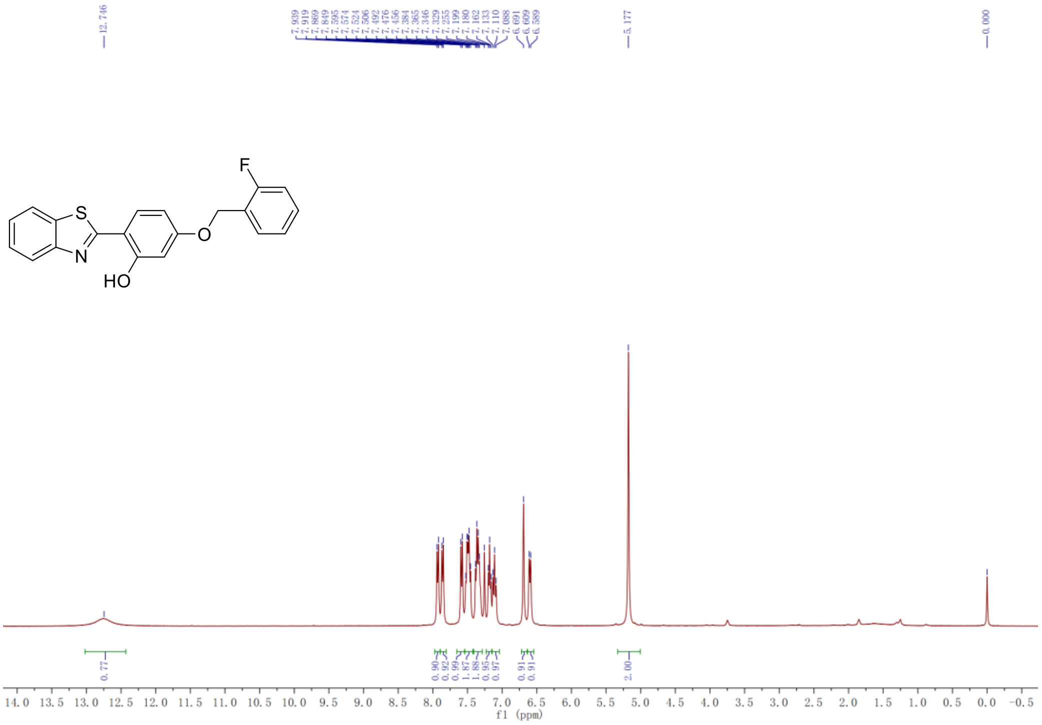

^{1}H NMR of compound 3b (400 MHz, $CDCl_3$)

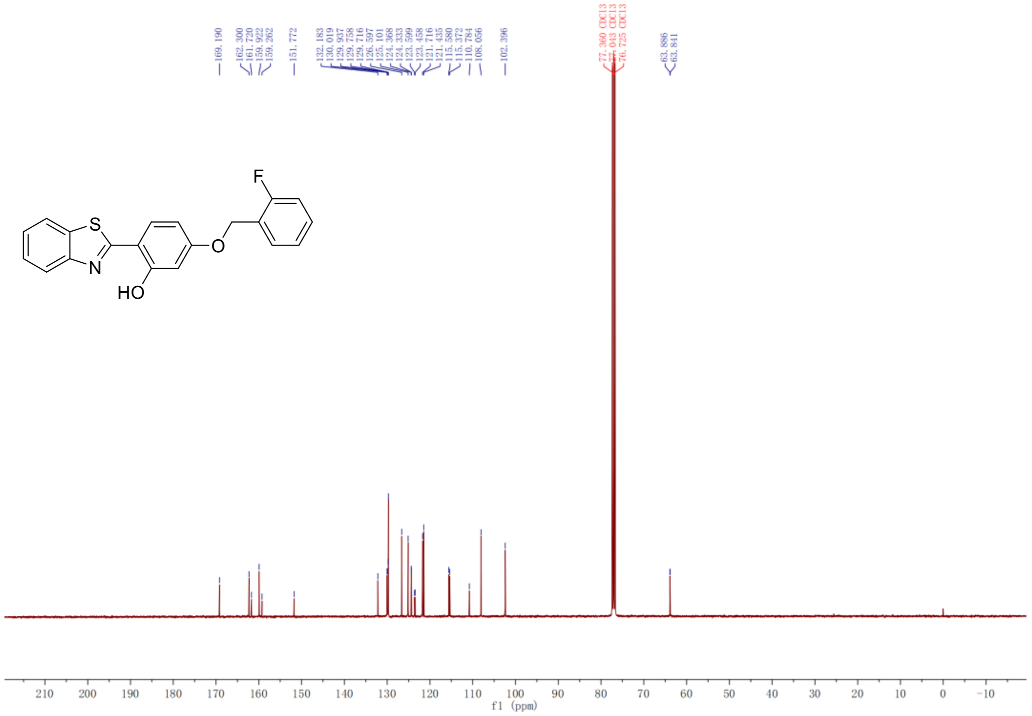

^{13}C NMR of compound 3b (100 MHz, $CDCl_3$)

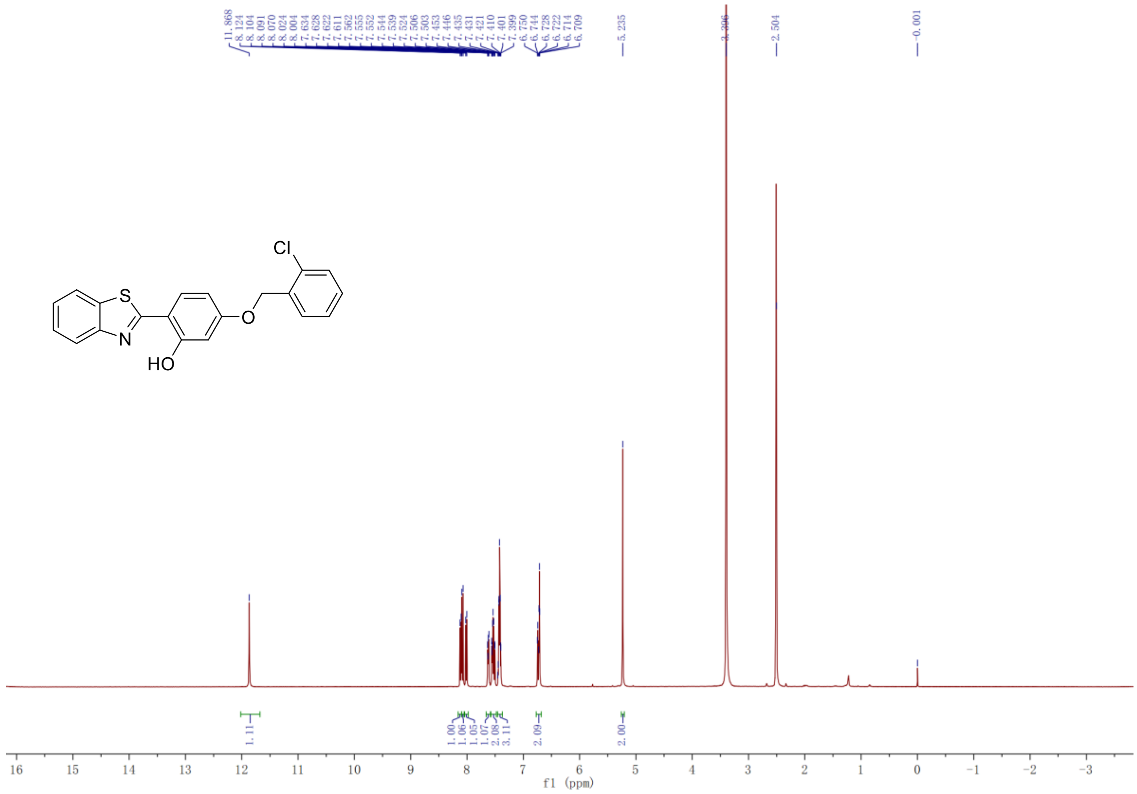

^{1}H NMR of compound 3c (400 MHz, DMSO-d_6)

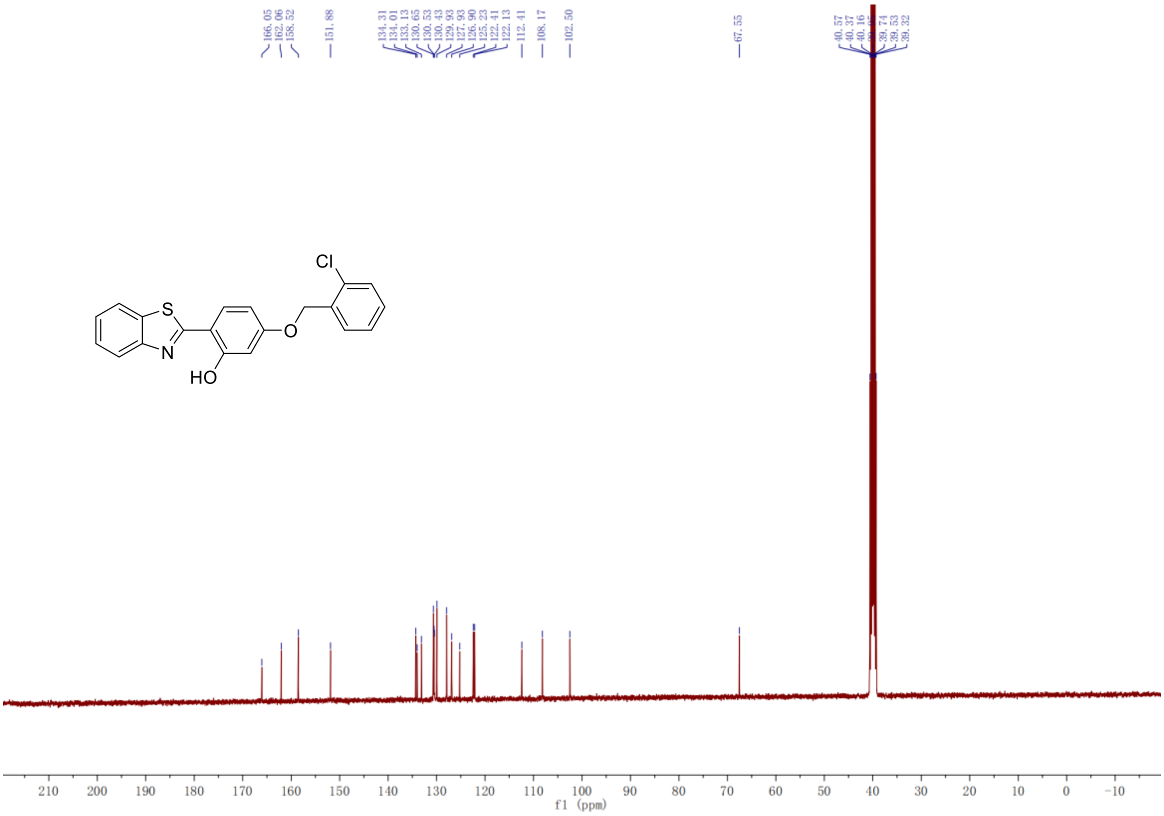

^{13}C NMR of compound 3c (100 MHz, DMSO-d_6)

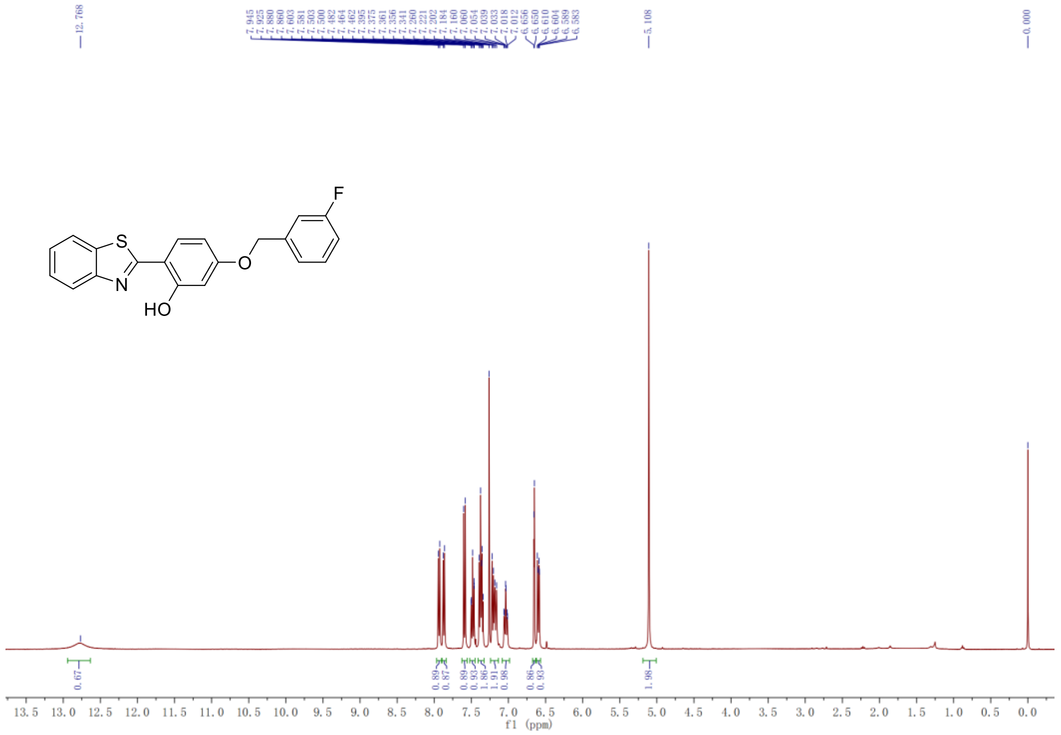

^{1}H NMR of compound 3d (400 MHz, $CDCl_3$)

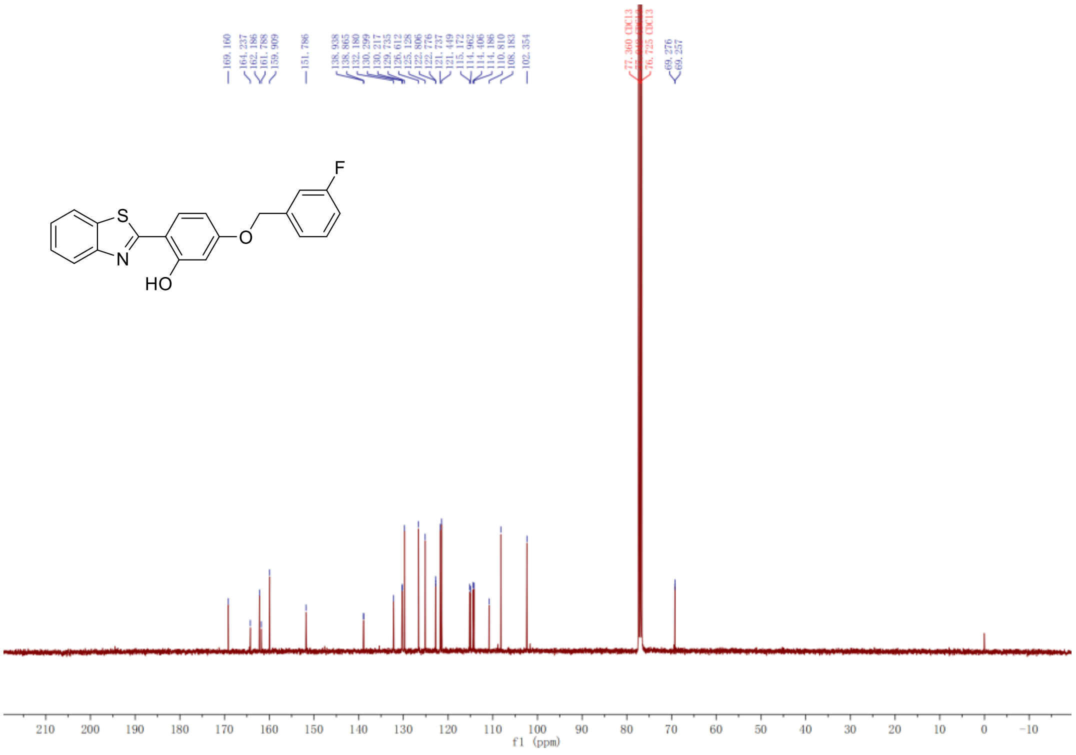

^{13}C NMR of compound 3d (100 MHz, $CDCl_3$)

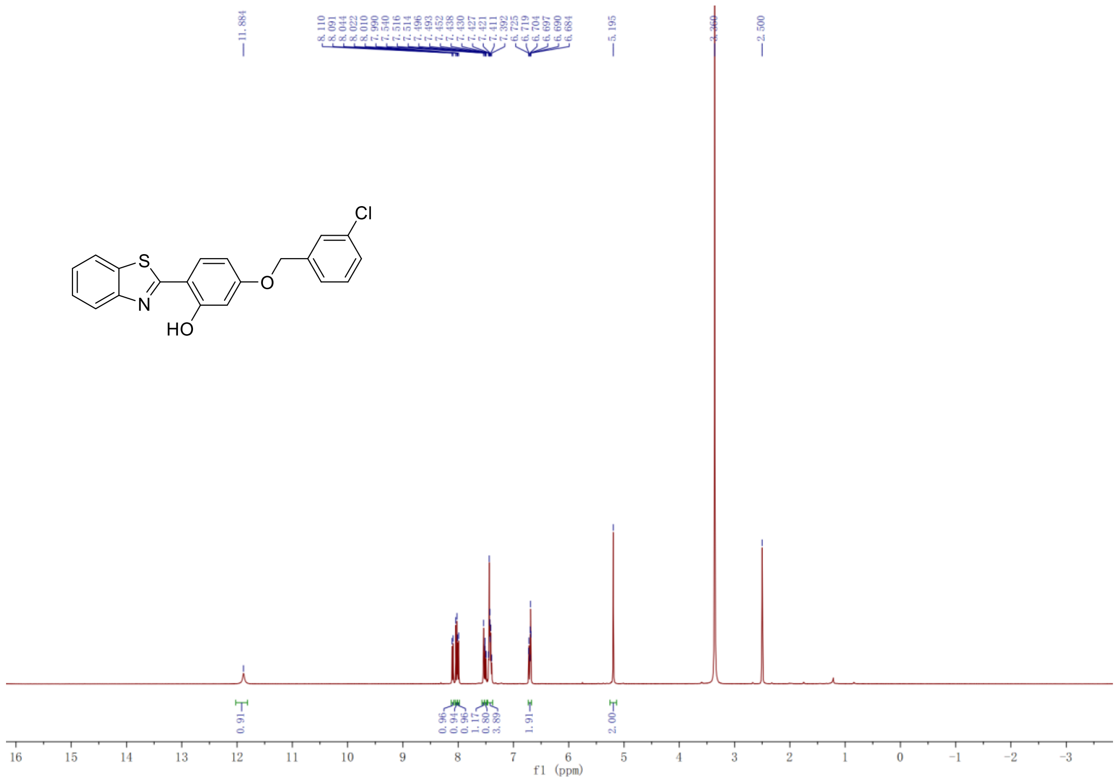

^{1}H NMR of compound 3e (400 MHz, DMSO-d_6)

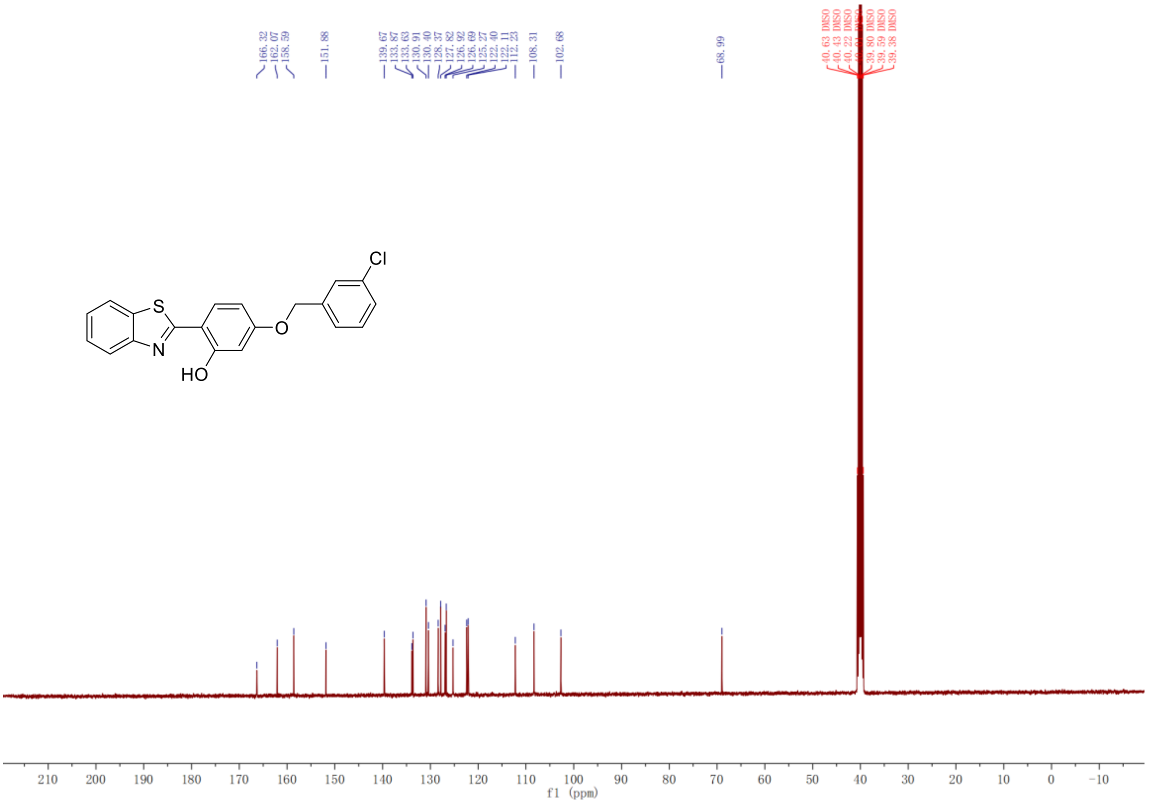

^{13}C NMR of compound 3e (100 MHz, DMSO-d_6)

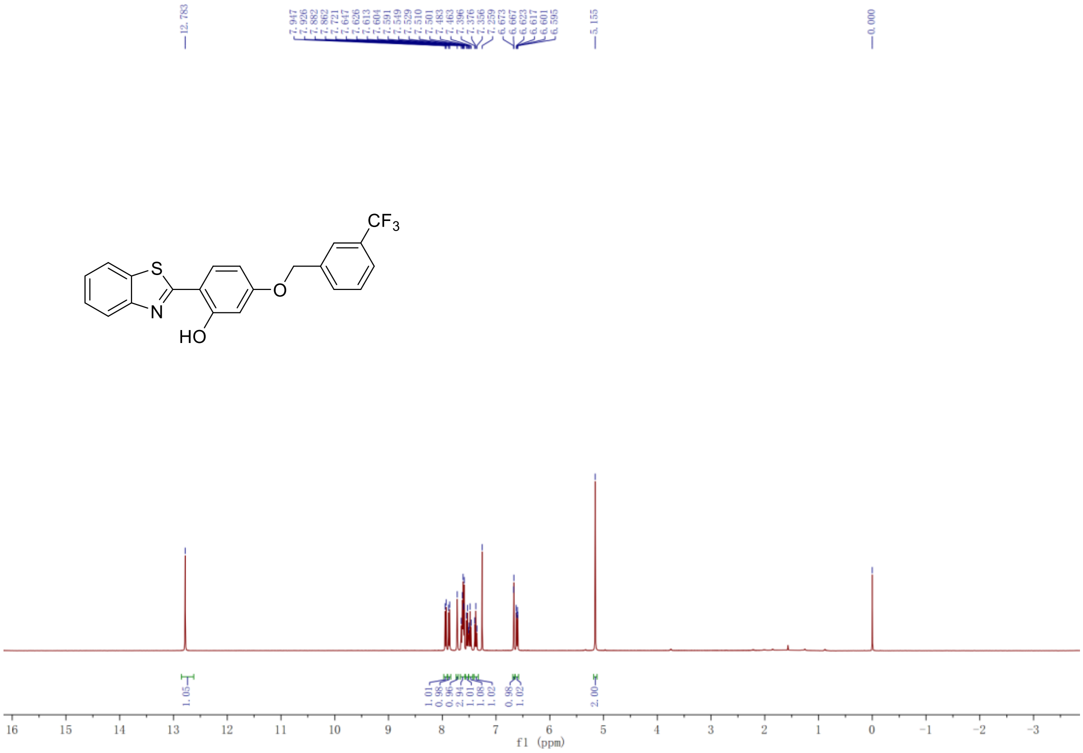

^{1}H NMR of compound 3f (400 MHz, $CDCl_3$)

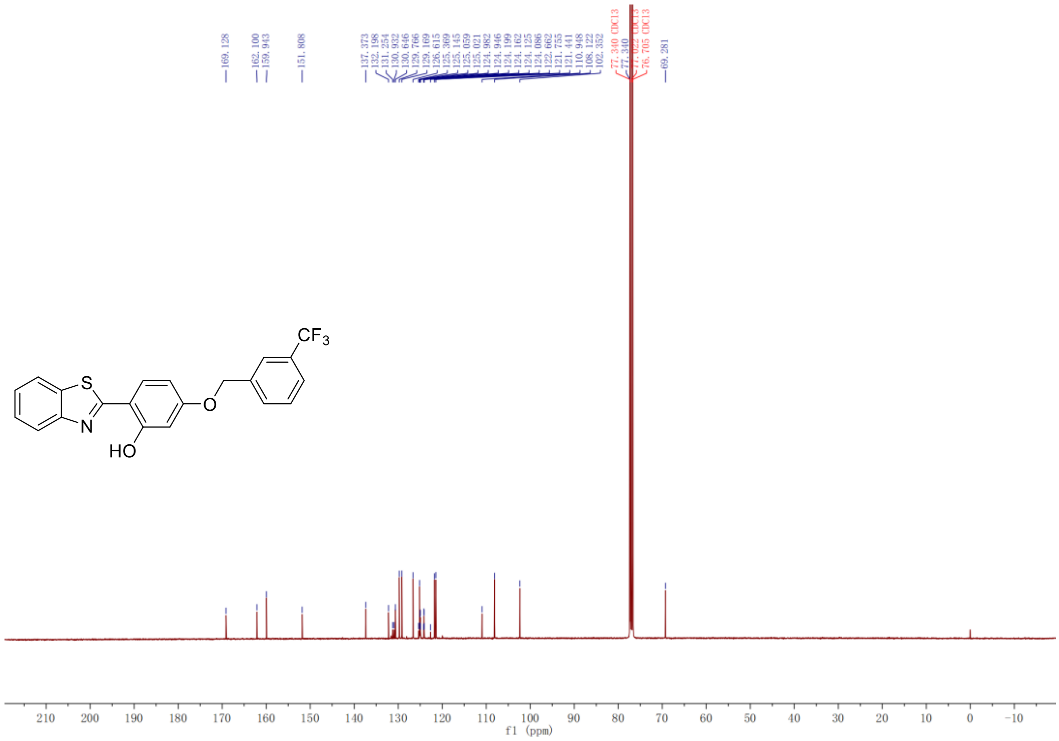

^{13}C NMR of compound 3f (100 MHz, $CDCl_3$)

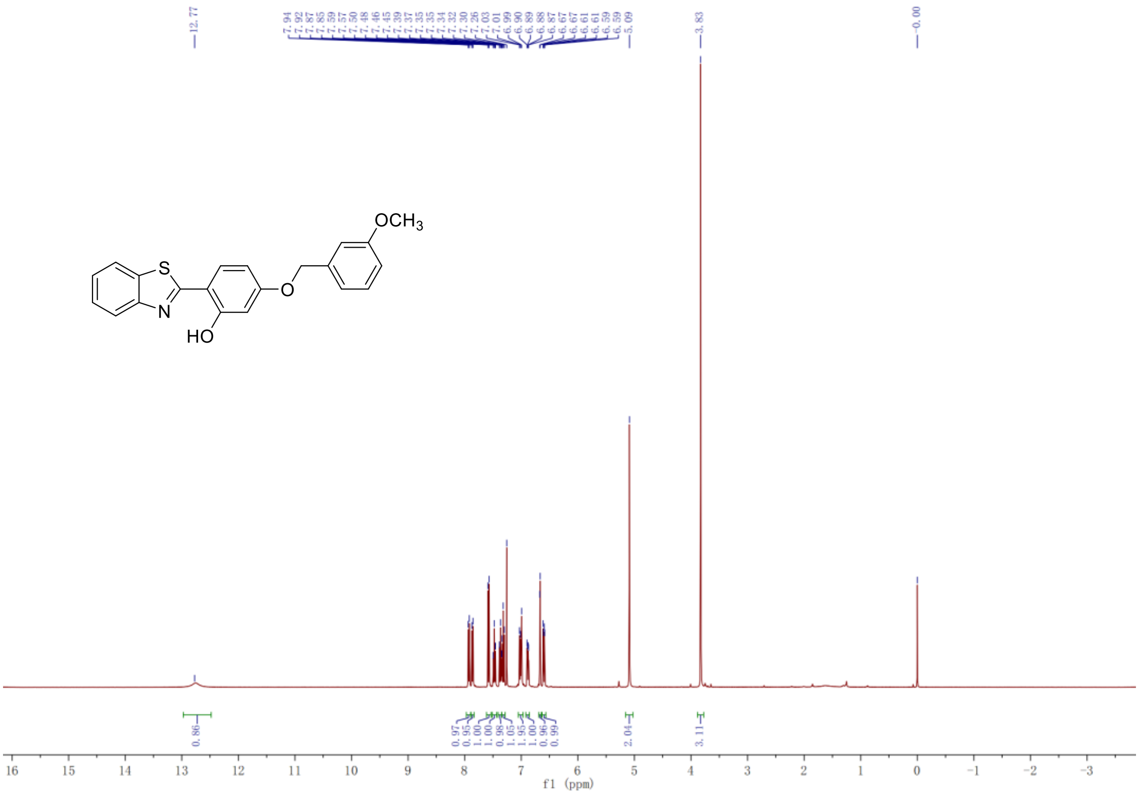

^{1}H NMR of compound 3g (400 MHz, $CDCl_3$)

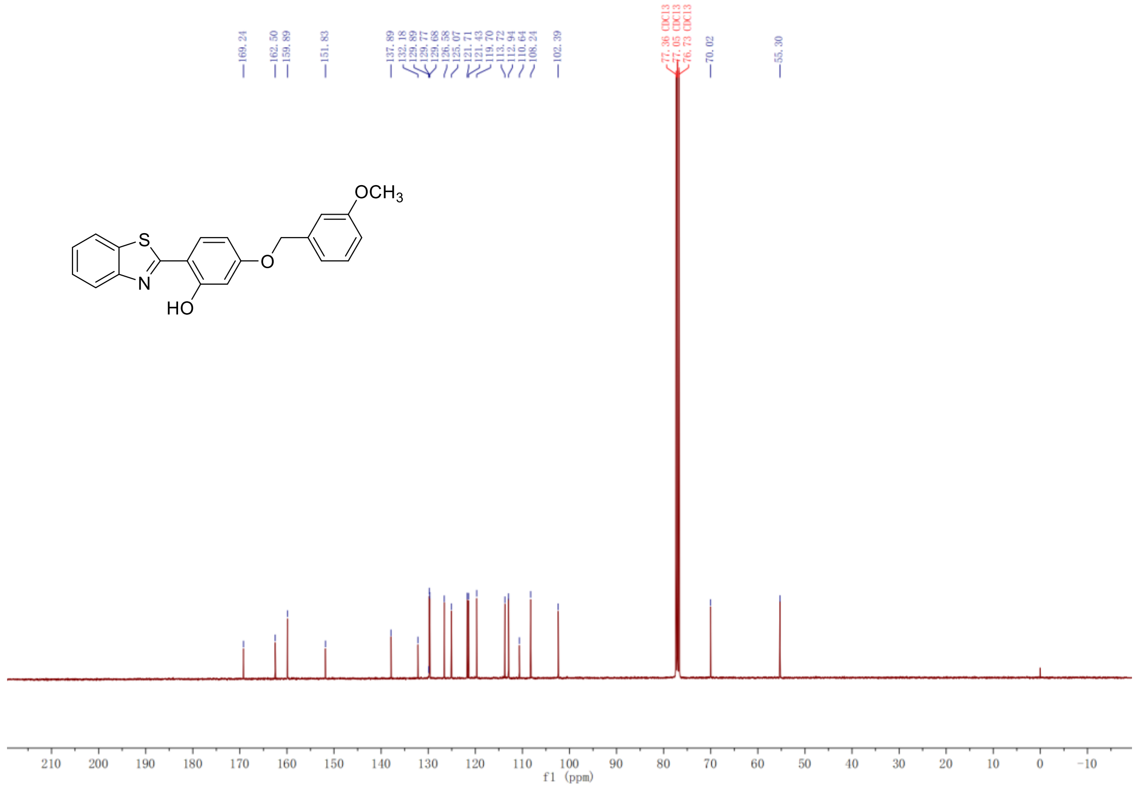

^{13}C NMR of compound 3g (100 MHz, $CDCl_3$)

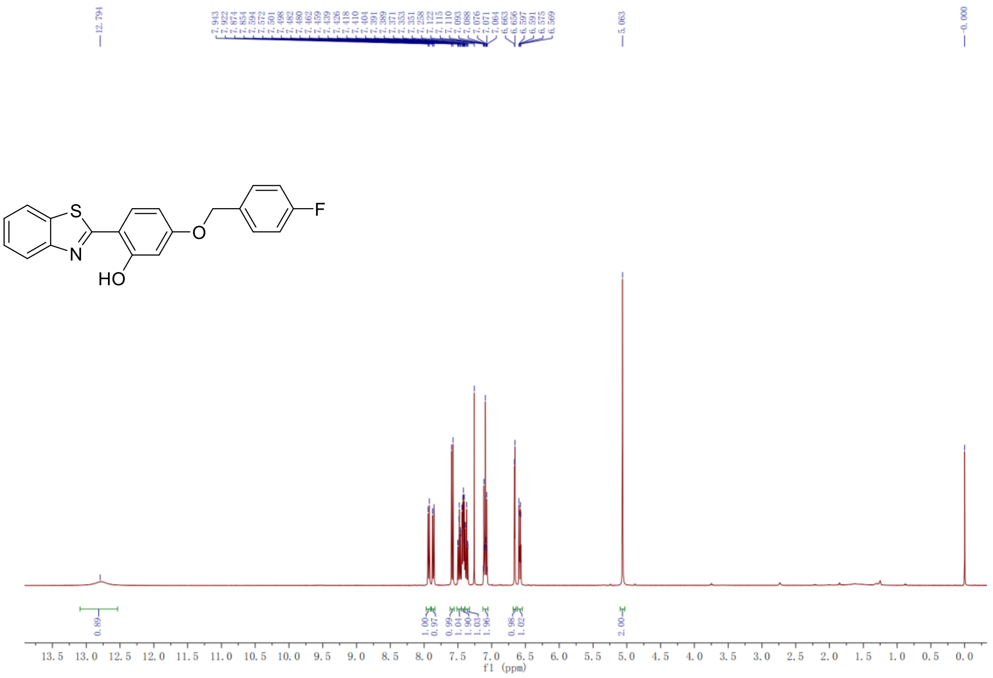

^{1}H NMR of compound 3h (400 MHz, $CDCl_3$)

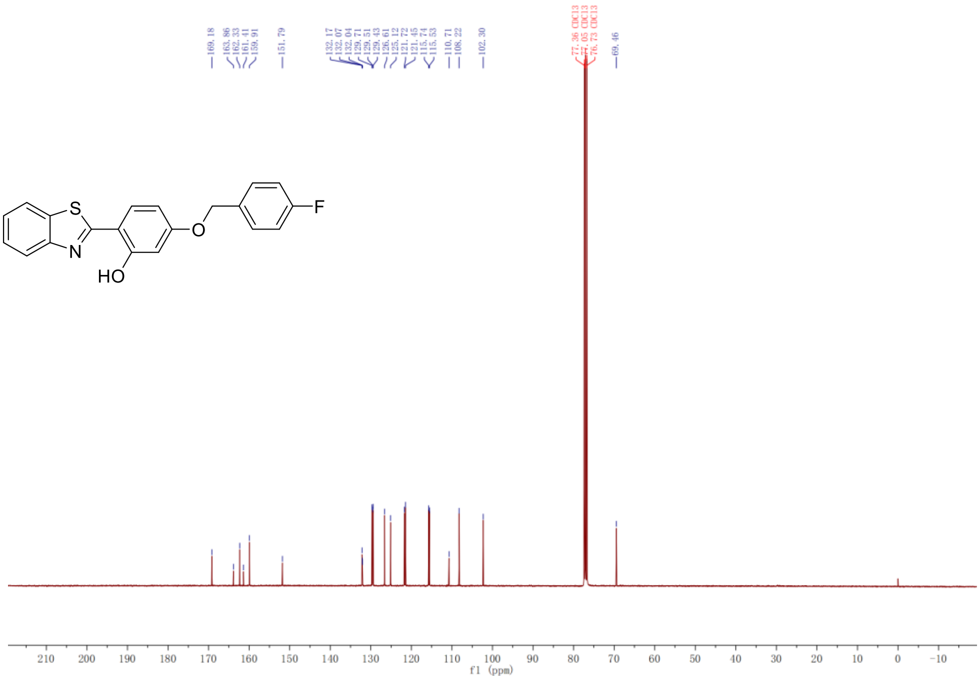

^{13}C NMR of compound 3h (100 MHz, $CDCl_3$)

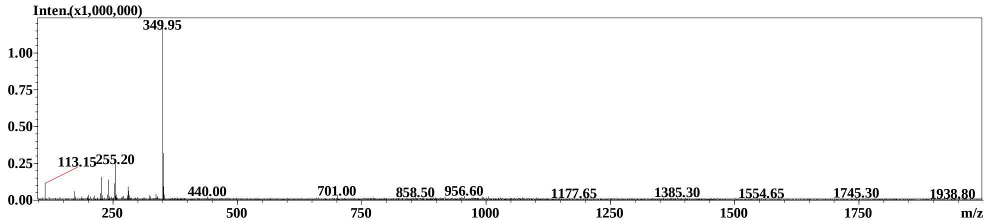

ESI-MS of compound 3h

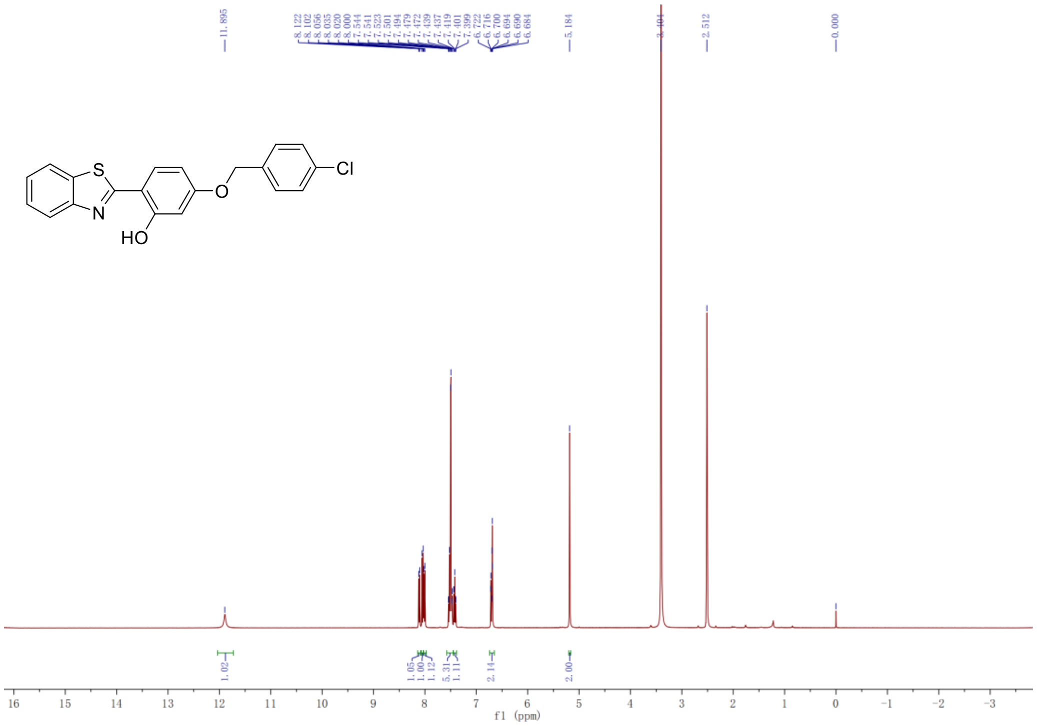

^{1}H NMR of compound 3i (400 MHz, DMSO-d_6)

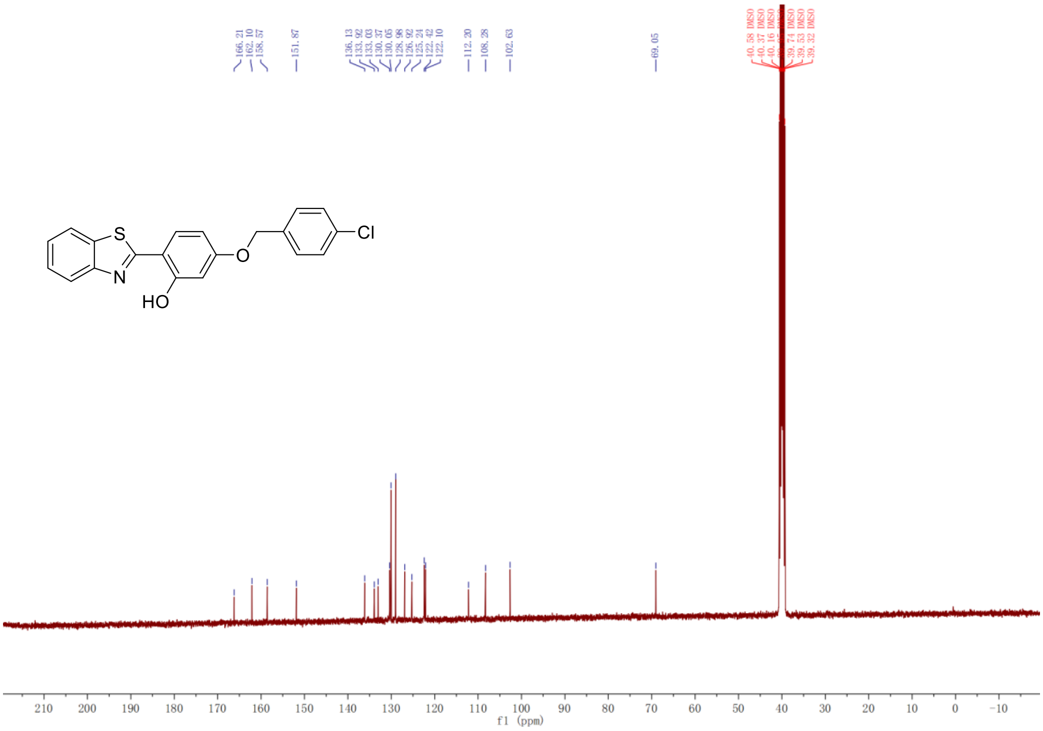

^{13}C NMR of compound 3i (100 MHz, DMSO-d_6)

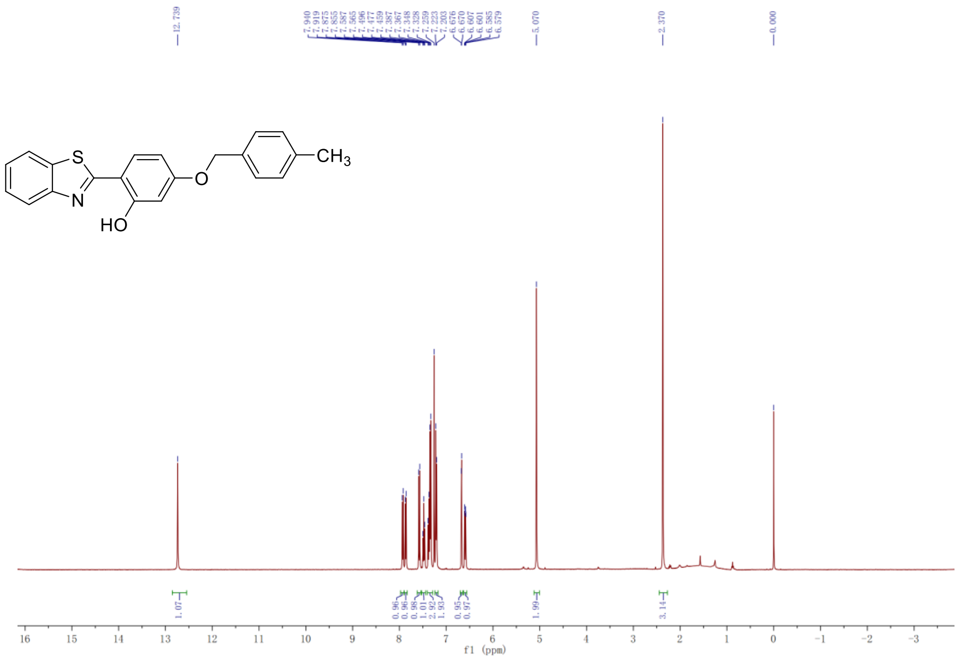

^{1}H NMR of compound 3j (400 MHz, $CDCl_3$)

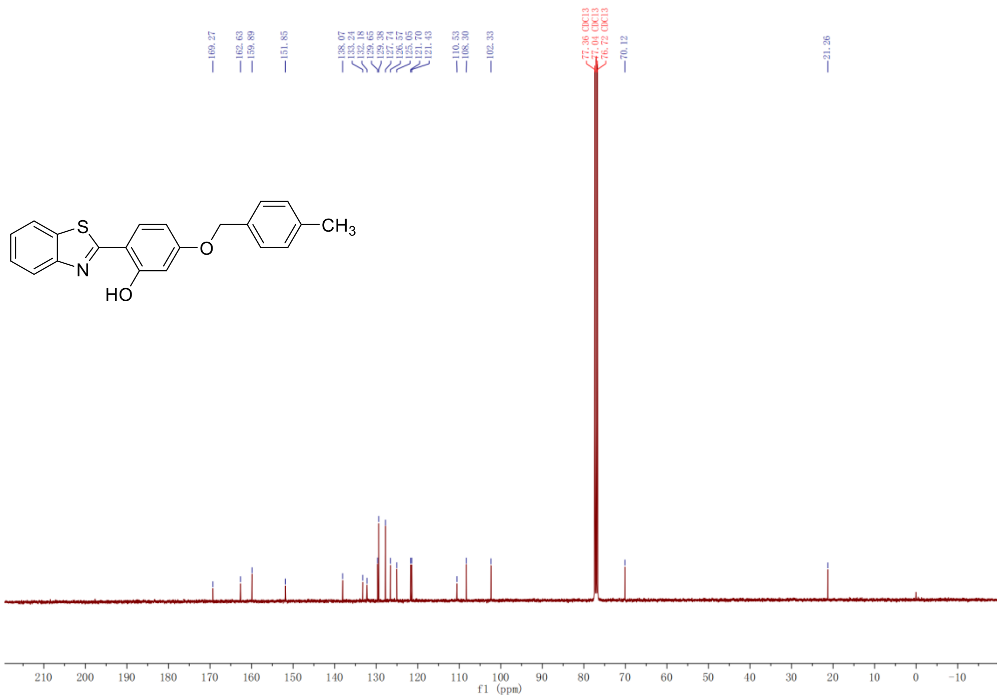

^{13}C NMR of compound 3j (100 MHz, $CDCl_3$)

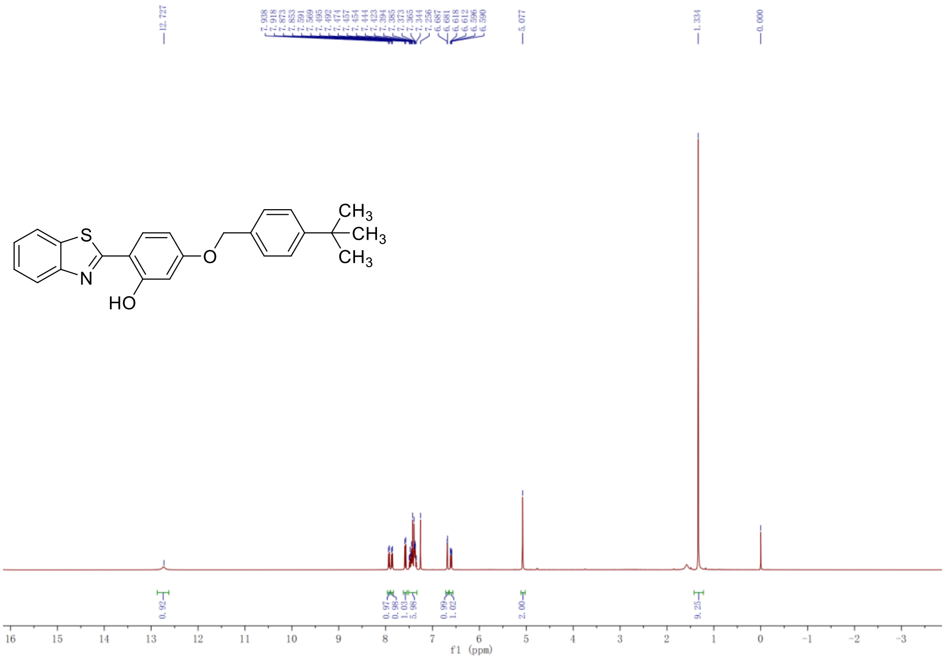

^{1}H NMR of compound 3k (400 MHz, $CDCl_3$)

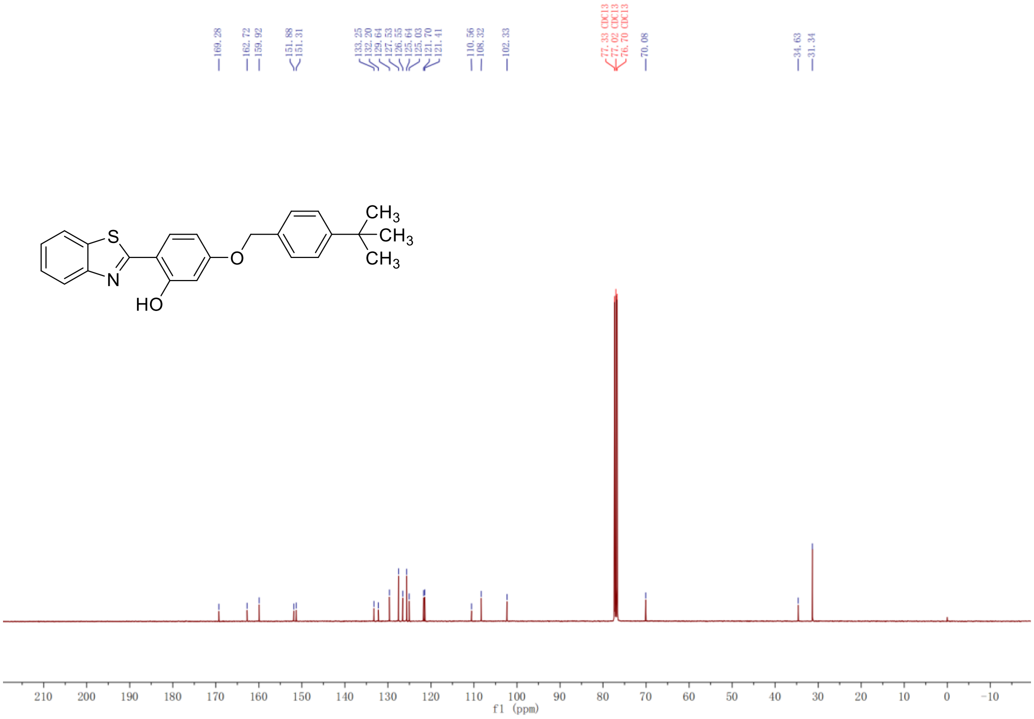

^{13}C NMR of compound 3k (100 MHz, $CDCl_3$)

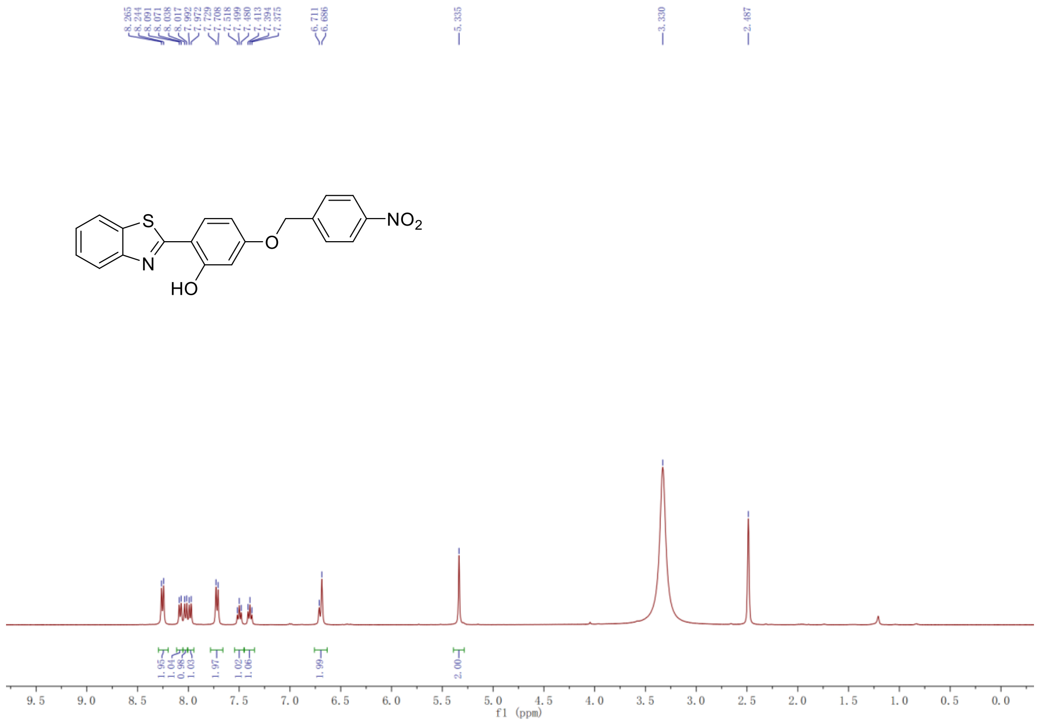

^{1}H NMR of compound 3l (400 MHz, DMSO-d_6)

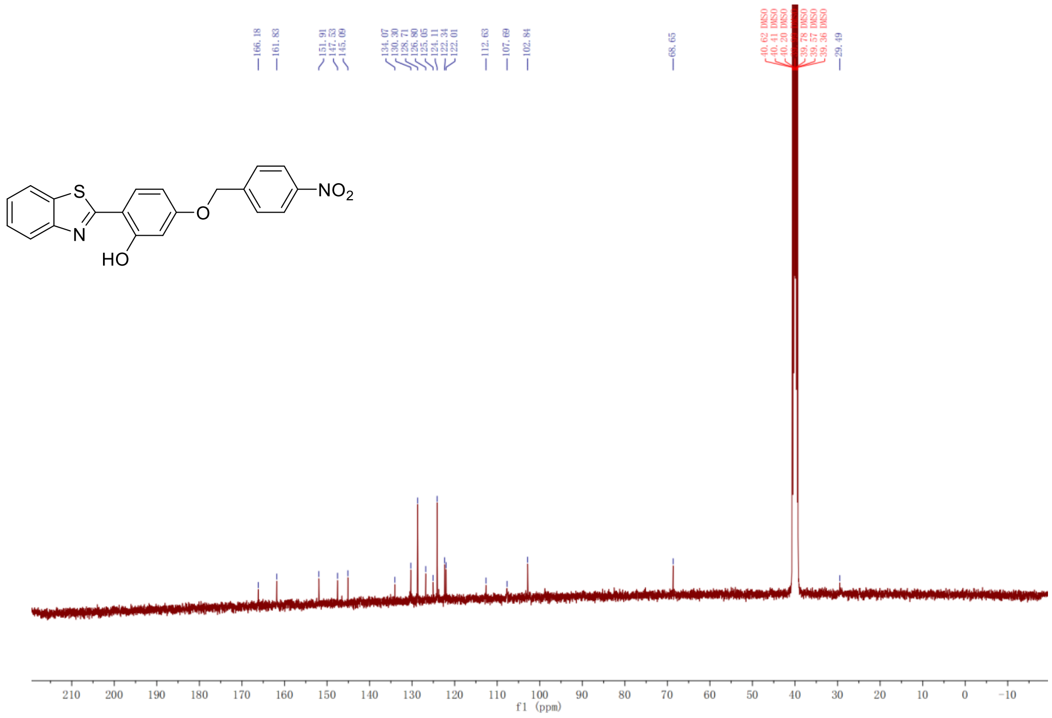

^{13}C NMR of compound 3l (100 MHz, DMSO-d_6)

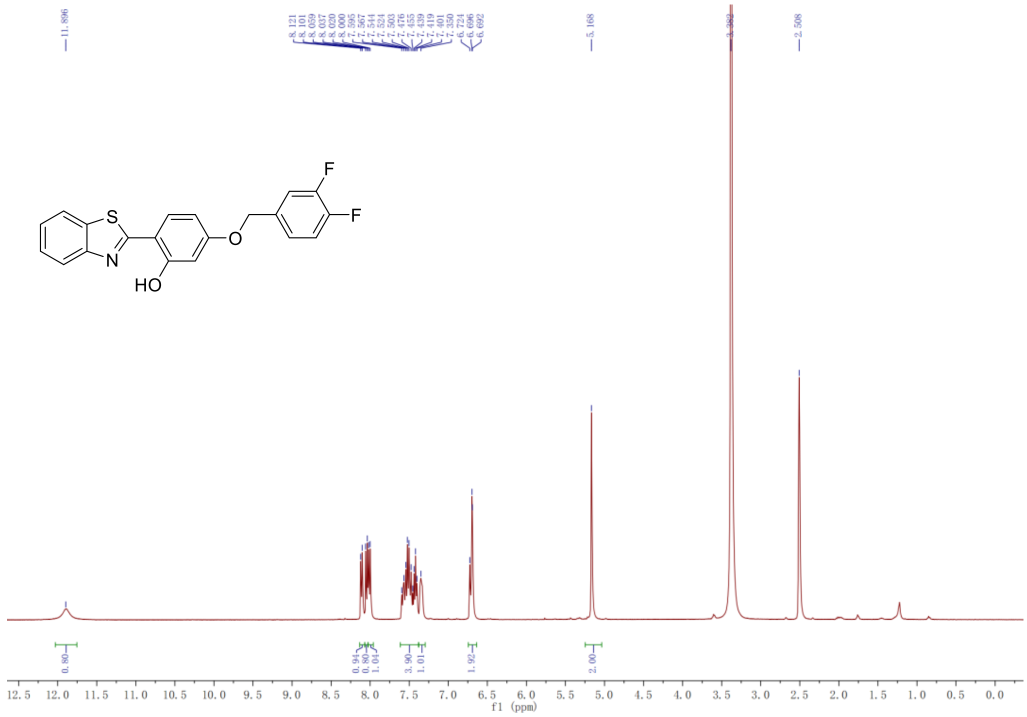

^{1}H NMR of compound 3m (400 MHz, DMSO-d_6)

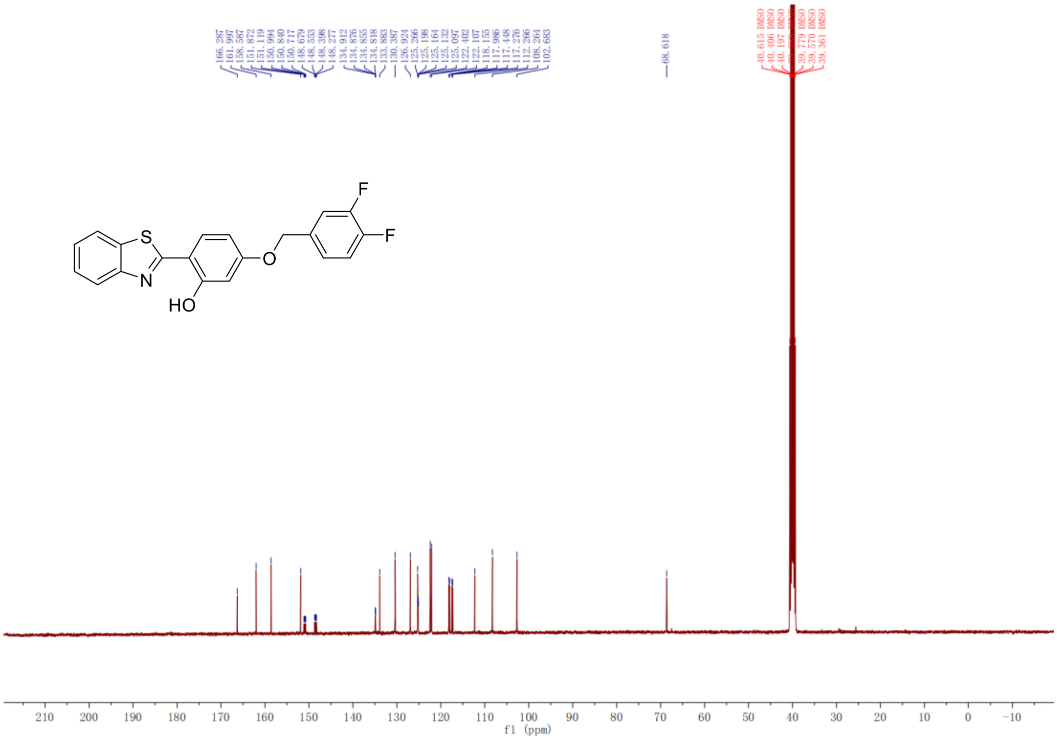

^{13}C NMR of compound 3m (100 MHz, DMSO-d_6)

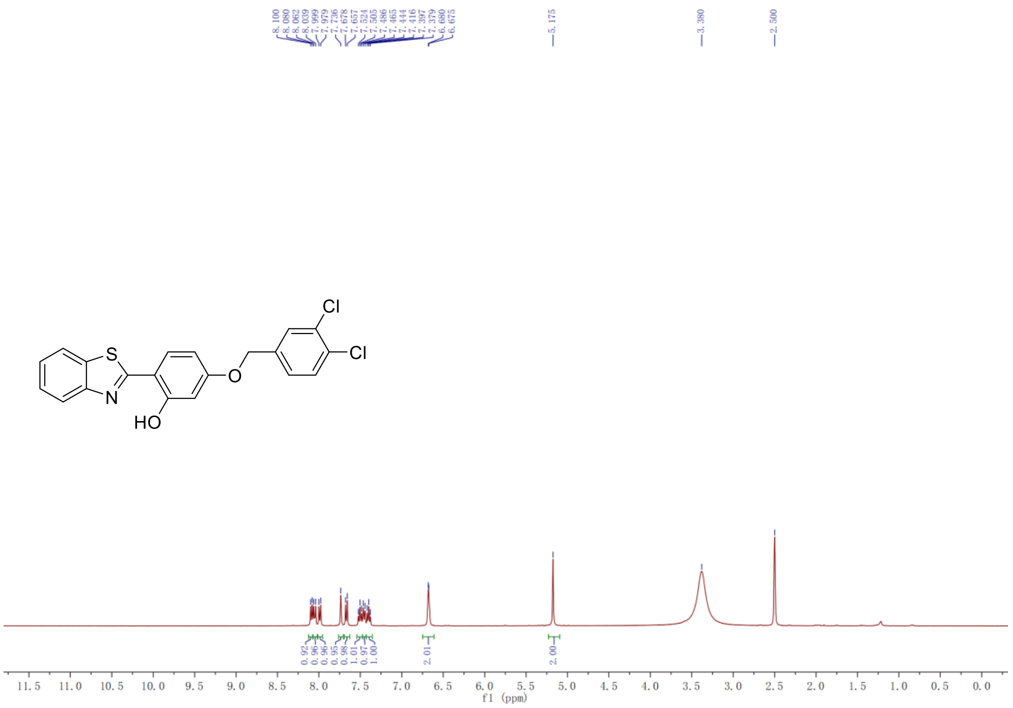

^{1}H NMR of compound 3n (400 MHz, DMSO-d_6)

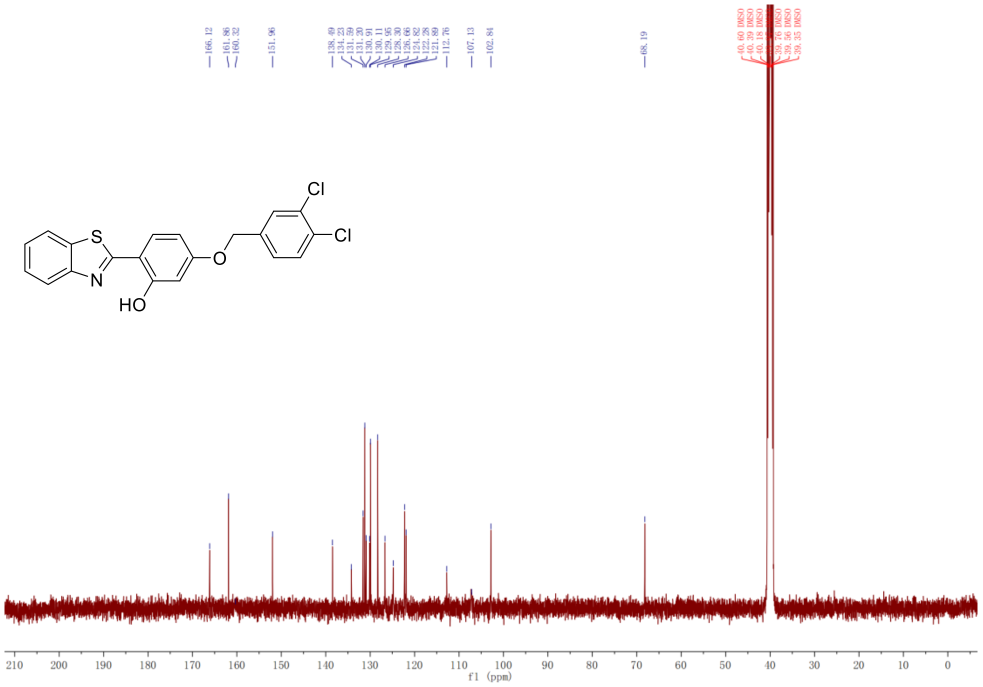

^{13}C NMR of compound 3n (100 MHz, DMSO-d_6)

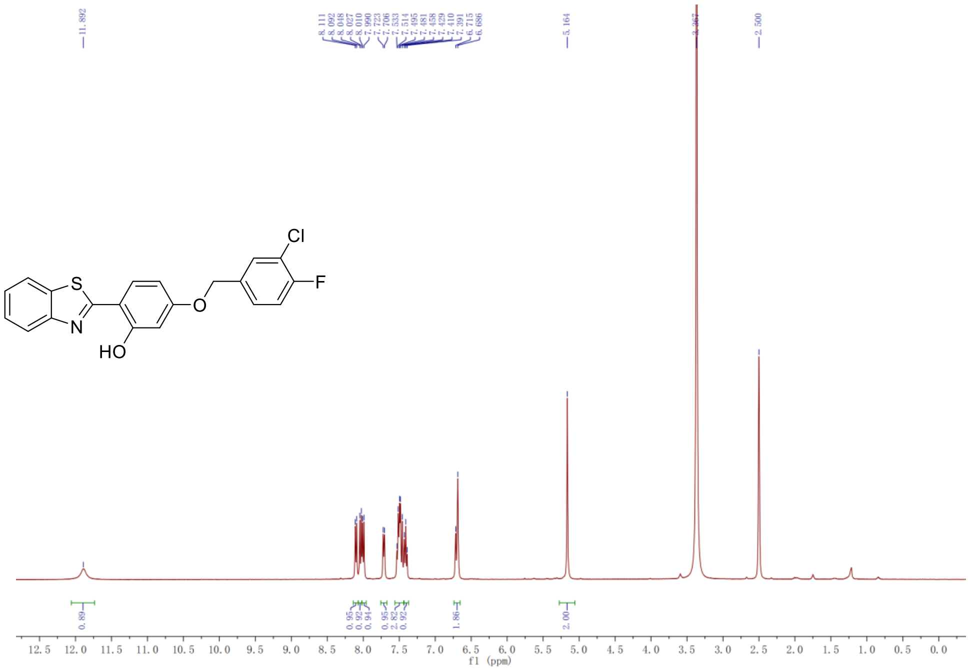

^{1}H NMR of compound 3o (400 MHz, DMSO-d_6)

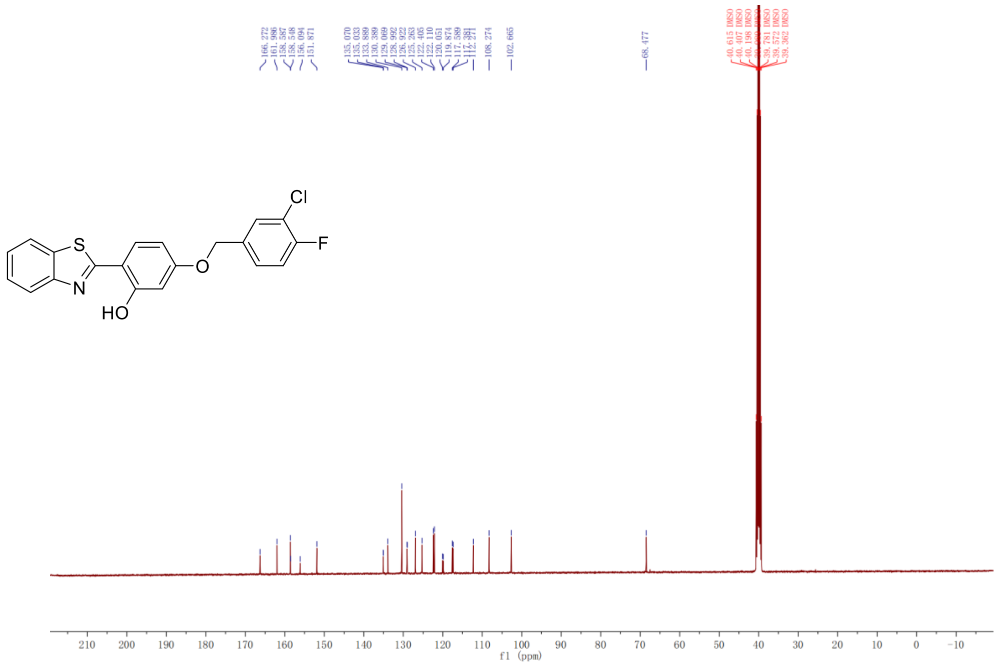

^{13}C NMR of compound 3o (100 MHz, DMSO-d_6)

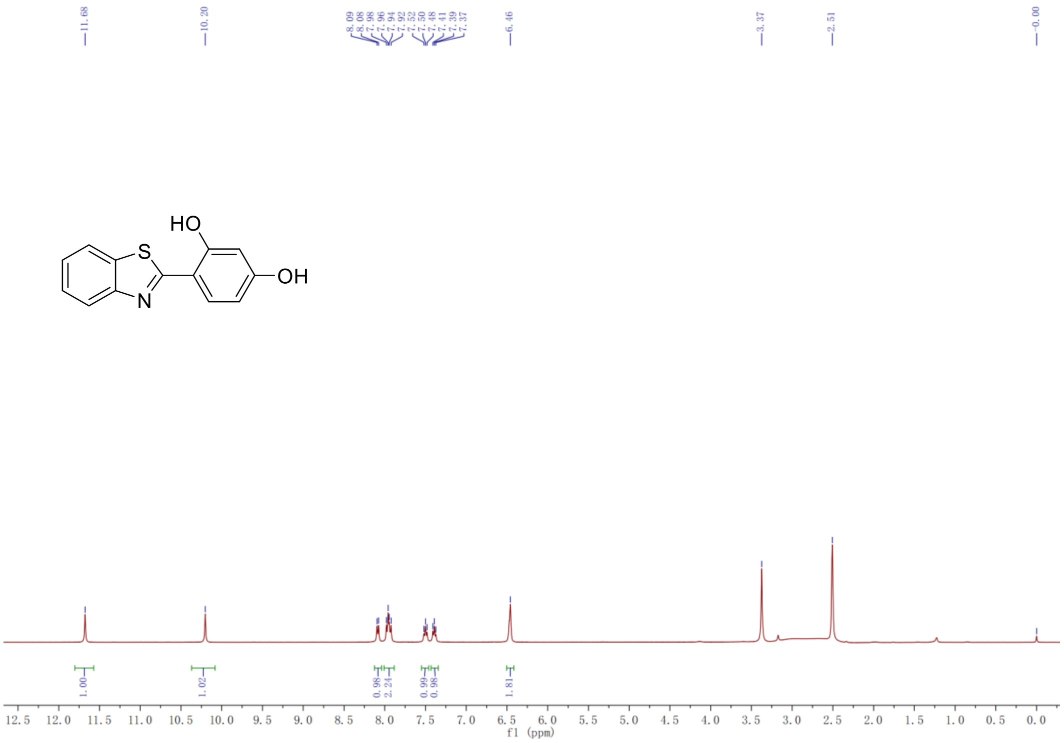

^{1}H NMR of compound 2 (400 MHz, DMSO-d_6)
